# SARS-CoV-2 promotes RIPK1 activation to facilitate viral propagation

**DOI:** 10.1038/s41422-021-00578-7

**Published:** 2021-10-18

**Authors:** Gang Xu, Ying Li, Shengyuan Zhang, Haoran Peng, Yunyun Wang, Dekang Li, Taijie Jin, Zhuohao He, Yilun Tong, Chunting Qi, Guowei Wu, Kangyun Dong, Jizhou Gou, Yang Liu, Tongyang Xiao, Jing Qu, Liang Li, Liang Liu, Ping Zhao, Zheng Zhang, Junying Yuan

**Affiliations:** 1 Institute of Hepatology, National Clinical Research Center for Infectious Disease, Shenzhen Third People’s Hospital, The Second Affiliated Hospital, School of Medicine, Southern University of Science and Technology, Shenzhen, Guangdong China; 2 Interdisciplinary Research Center on Biology and Chemistry, Shanghai Institute of Organic Chemistry, Chinese Academy of Sciences, 100 Haike Rd, Pudong, Shanghai, China; 3 Department of Microbiology, Second Military Medical University, 800 Xiangyin Rd, Shanghai, China; 4 Department of Forensic Medicine, Tongji Medical College, Huazhong University of Science and Technology, Wuhan, Hubei China; 5 University of Chinese Academy of Sciences, Beijing, China; 6 Department for Pathology, Shenzhen Third People’s Hospital, Shenzhen, Guangdong China; 7 Institute of Biomedicine and Biotechnology, Shenzhen Institutes of Advanced Technology, Chinese Academy of Sciences, Nanshan, Shenzhen, Guangdong China

**Keywords:** Cell biology, Mechanisms of disease

## Abstract

Coronavirus disease 2019 (COVID-19), caused by severe acute respiratory syndrome coronavirus 2 (SARS-CoV-2), is the ongoing global pandemic that poses substantial challenges to public health worldwide. A subset of COVID-19 patients experience systemic inflammatory response, known as cytokine storm, which may lead to death. Receptor-interacting serine/threonine-protein kinase 1 (RIPK1) is an important mediator of inflammation and cell death. Here, we examined the interaction of RIPK1-mediated innate immunity with SARS-CoV-2 infection. We found evidence of RIPK1 activation in human COVID-19 lung pathological samples, and cultured human lung organoids and ACE2 transgenic mice infected by SARS-CoV-2. Inhibition of RIPK1 using multiple small-molecule inhibitors reduced the viral load of SARS-CoV-2 in human lung organoids. Furthermore, therapeutic dosing of the RIPK1 inhibitor Nec-1s reduced mortality and lung viral load, and blocked the CNS manifestation of SARS-CoV-2 in ACE2 transgenic mice. Mechanistically, we found that the RNA-dependent RNA polymerase of SARS-CoV-2, NSP12, a highly conserved central component of coronaviral replication and transcription machinery, promoted the activation of RIPK1. Furthermore, NSP12 323L variant, encoded by the SARS-CoV-2 C14408T variant first detected in Lombardy, Italy, that carries a Pro323Leu amino acid substitution in NSP12, showed increased ability to activate RIPK1. Inhibition of RIPK1 downregulated the transcriptional induction of proinflammatory cytokines and host factors including ACE2 and EGFR that promote viral entry into cells. Our results suggest that SARS-CoV-2 may have an unexpected and unusual ability to hijack the RIPK1-mediated host defense response to promote its own propagation and that inhibition of RIPK1 may provide a therapeutic option for the treatment of COVID-19.

## Introduction

Coronavirus disease 2019 (COVID-19), caused by severe acute respiratory syndrome coronavirus 2 (SARS-CoV-2),^1^ is the ongoing global pandemic that poses substantial challenges to public health worldwide. Angiotensin-converting enzyme 2 (ACE2) is the major recognized cell-surface receptor enabling cellular entry of SARS-CoV-2.^2^ A subset of COVID-19 patients experience systemic inflammatory response, known as cytokine storm, which may lead to a severe disease with multi-organ damage and eventually be fatal.^3,4^ While the importance of an effective management for inflammation in the treatment of severe COVID-19 is known,^5^ the interaction of SARS-CoV-2 with host innate immune system and in particular, the significance of systemic inflammatory response to the propagation of SARS-CoV-2 is not well understood. In addition, the neurological implication of COVID-19 has been increasingly recognized.^6^ SARS-CoV-2 can enter the nervous system by crossing the neural–mucosal interface in olfactory mucosa.^7^ A retrospective analysis of 214 COVID-19 patients in Wuhan, China, found that 36.4% of patients developed neurological symptoms, such as dizziness and headache, in the early stages; and furthermore, neurological symptoms are significantly more common in severe patients than in non-severe patients.^8^ An electronic health record analysis of 81 million patients in the United States found that COVID-19 patients had a 44% higher risk of developing a neuropsychiatric disease than that of flu patients.^9^ However, it is currently unclear how to effectively control the CNS manifestation of this disease.

The genetic variants of SARS-CoV-2 present serious challenges in combating this ongoing pandemic. The SARS-CoV-2 C14408T variant that carries a Pro323Leu amino acid substitution in RNA-dependent RNA polymerase (RdRp, NSP12), the central component of coronaviral replication and transcription machinery, was first detected in the early outbreak phase in Lombardy, Italy and quickly established as the dominant SARS-CoV-2 variant detected in between 50% and 70% of SARS-CoV-2 sequences around the world, particularly in Europe, North and South America, and Africa, with significantly higher infectivity and transmissibility than the original variant.^10–12^ In addition, the SARS-CoV-2 viral variants carrying P323L mutation in NSP12 and the associated D614G mutation in spike protein were reported to be enriched in severely affected group.^13^ The mechanism behind the apparent positive selection of the variant with C14408T mutation is unclear with most studies focused on the Spike protein.

Receptor-Interacting Serine/Threonine-Protein Kinase 1 (RIPK1) is an important mediator of inflammation and cell death.^14^ Activation of RIPK1 is known to mediate innate immune responses such as apoptosis, necroptosis and inflammation.^15^ The activation of RIPK1 downstream of TNFR1 signaling can promote apoptosis by mediating the activation of caspase-8 or necroptosis by mediating the activation of RIPK3 and MLKL.^16–18^ Importantly, the activation of RIPK1 kinase has been increasingly recognized to play an important role in promoting inflammation, including neurodegenerative diseases involving CNS.^19–21^ RIPK1 activation was found in the upper respiratory epithelial cells of COVID-19 patients.^22^ In addition, in silico bioinformatic analyses of multiple datasets on SARS-CoV-2-infected cells and patient samples led to the suggestion that RIPK1 may be an important target for the treatment of COVID-19.^23,24^ However, the effect or the mechanism of inhibiting RIPK1 in cells and animal models of SARS-CoV-2 has not yet been examined.

Here, we examined the role of RIPK1 in SARS-CoV-2 infection using human COVID-19 lung pathological samples, cultured human lung organoids and ACE2 transgenic mice. The activation of RIPK1 was found in human COVID-19 pathological samples, and human lung organoids and ACE2 transgenic mice infected by SARS-CoV-2. Inhibition of RIPK1 using multiple small-molecule inhibitors of RIPK1 reduced the proliferation of SARS-CoV-2 in human lung organoids in culture. We found that the RdRp of SARS-CoV-2 (NSP12) promoted the activation of RIPK1. Inhibition of RIPK1 by small-molecule inhibitor Nec-1s in human lung organoids infected by SARS-CoV-2 expressing NSP12 323P and 323L variants downregulated the transcriptional induction of proinflammatory cytokines and host factors ACE2 and the epidermal growth factor receptor (EGFR) that mediate viral entry, and reduced viral load. Finally, therapeutic treatment with Nec-1s reduced mortality, lung viral load and CNS manifestation in vivo in ACE2 transgenic mice infected with SARS-CoV-2, a severe COVID-19 animal model. Our results suggest that SARS-CoV-2 may have an unexpected and unusual ability to hijack the RIPK1-mediated host defense response to promote its own propagation and that inhibition of RIPK1 may provide a therapeutic option for the treatment of severe COVID-19.

## Results

### Activation of RIPK1 in COVID-19 lungs

To explore the mechanism of dysregulated innate immune response in COVID-19, we compared the proinflammatory cytokine transcriptomes in the bronchoalveolar lavage fluid (BALF), peripheral blood mononuclear cells (PBMCs) and lung tissue from the same patient with severe COVID-19 using single-cell RNA sequencing (scRNA-seq) (Supplementary information Fig. S1a, b). We found upregulation of proinflammatory cytokines consistent with previous findings in severe COVID-19.^25^ By comparing cytokine profiles in the PBMC, lung and BALF samples, we found that levels of proinflammatory cytokine transcripts, such as interleukin 6 (IL6), tumor necrosis factor (TNF), CXC-chemokine ligand 10 (CXCL10), CC-chemokine ligand 2 (CCL2, also called MCP-1 or monocyte chemotactic protein 1), CCL3 and CCL4, were much higher in the BALF and lung samples than in the PBMC sample, which is consistent with the pathological focus of severe COVID-19 in the lung parenchyma.^26^

**Fig. 1:**
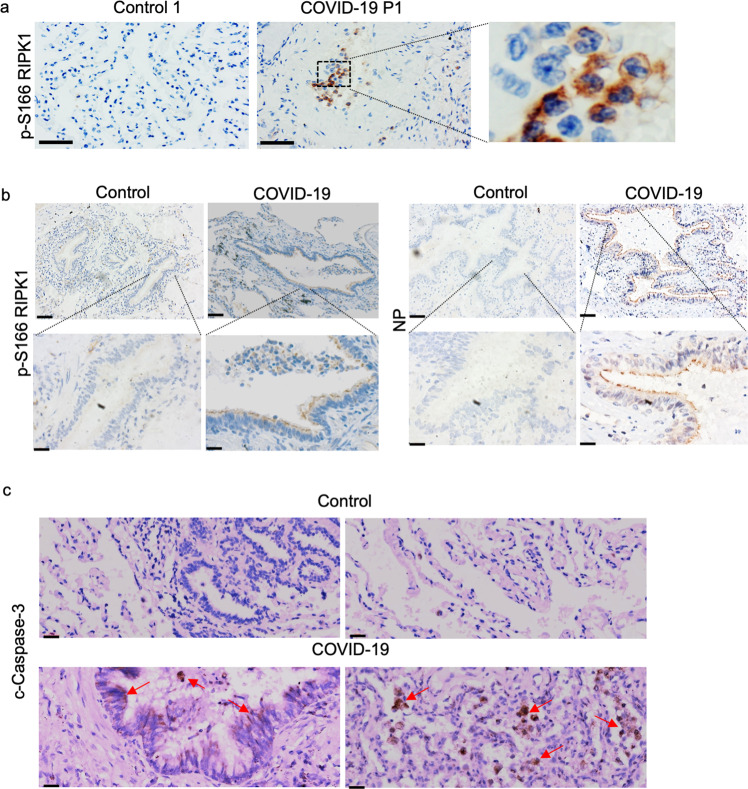
RIPK1 was activated in the COVID-19 lungs. **a** IHC staining of p-S166 RIPK1 in the lungs of a COVID-19 patient (middle and right) and an age-matched control (left). Scale bars, 1 inch. The enlarged image (right) shows the nuclear morphology of p-S166 RIPK1^+^ cells. **b** Activated RIPK1 in the bronchial ciliated epithelial cells of patients with severe COVID-19. IHC staining of viral nucleocapsid protein (NP) and p-S166 RIPK1 in the bronchial region of the COVID-19 patient and control was shown. The lower row shows the enlarged images of specified areas above. Scale bars, 100 μm. **c** IHC staining of c-casp3 in the lungs of patients with severe COVID-19 (bottom) and control (top). Scale bars, 20 μm.

Activation of RIPK1 in human neurodegenerative and inflammatory diseases has been shown to promote the production of proinflammatory cytokines such as IL6 and TNF.^19–21^ This is also seen in patients with mutations that activate RIPK1 by blocking the caspase-8 cleavage site in RIPK1.^14,27,28^ To explore the involvement of RIPK1, we examined autopsied lung tissues from 4 deceased severe COVID-19 patients and age-matched control lung samples (Supplementary information Table S1) for evidence of RIPK1 activation using anti-p-S166 RIPK1, a biomarker of RIPK1 activation.^20,29^ Major pathological features of COVID-19 lungs include extensive alveolar damage, accumulation of macrophages and neutrophils in the alveolar cavities and inflammatory exudation in the alveoli and interstitial tissues.^30^ Activated RIPK1 was found in a substantial number of macrophages (CD68^+^) and neutrophils (CD66B^+^) within the alveolar cavities (Fig. 1a and Supplementary information Fig. S2a). In contrast, activated RIPK1 was only found in sparsely distributed macrophages in control human lung samples. Activated RIPK1 is known to mediate inflammation in the cells of myeloid lineage, including macrophages and neutrophils.^14,31,32^ Thus, inflammatory cytokine storm in COVID-19 patients’ lung is associated with activation of RIPK1 in macrophages and neutrophils.

We found a strikingly enriched presence of activated RIPK1 in the ciliated epithelial cells in the airways of severe COVID-19 patients which corresponds to elevated levels of proinflammatory cytokines and factors in the BALF, lung and PBMCs in patients compared to that of control individuals (Fig. 1b and Supplementary information Figs. S1b and S2b). The ciliated epithelial cells line the extensive surface of the respiratory tract between the nose and the alveoli in the lung and are heavily exposed to viral and bacterial pathogens.^33^ Ciliated epithelial cells in the nose and conducting airways, the bronchi and bronchioles, have been found to be the cell type that most frequently expresses the SARS-CoV-2 entry receptor ACE2 and the serine protease TMPRSS2, and are susceptible for SARS-CoV-2 infection in pathological lung samples of COVID-19 patients and in lung culture studies.^34–37^ SARS-CoV-2 nucleocapsid protein (NP) was found in the epithelial cells, including ciliated epithelial cells, of bronchial and bronchiolar airways.^38^ Using immunohistochemistry (IHC) staining, we found an enriched presence of SARS-CoV-2 NP in ciliated epithelial cells in the COVID-19-positive lung, which corresponds to the ciliated epithelial cells with activated RIPK1 (Fig. 1b and Supplementary information Fig. S2c). RIPK1 is expressed in epithelial cells and macrophages in both control and COVID-19-positive lungs (Supplementary information Fig. S3a, b). In addition, the increase of RIPK1^+^ cells in severe COVID-19 lungs is in part due to infiltrating macrophages and neutrophils which express high levels of RIPK1.

Since activation of RIPK1 can promote necroptosis, apoptosis and inflammation,^39^ we investigated each of these three possibilities by examining their biomarkers in COVID-19 lungs. We found that the expression of RIPK3 was only detectable by IHC in a few macrophages and p-RIPK3 was undetectable in COVID-19-positive lung (Supplementary information Fig. S3c). The mRNA levels of *RIPK3* in lung epithelial cells were also low, mostly limited to inflammatory macrophages (Supplementary information Fig. S3d). On the other hand, the expression of MLKL was highly upregulated in the severe COVID-19 lung and was detected in lung and airway epithelial cells as well as macrophages and neutrophils (Supplementary information Fig. S4a, b). However, no p-MLKL was detected in the COVID-19 lung (Supplementary information Fig. S4c). Since the phosphorylation of MLKL is mediated by RIPK3 in necroptosis,^40^ the low-level expression of RIPK3 in COVID-19 lung may preclude the activation of MLKL downstream of activated RIPK1 to promote necroptosis. Since MLKL is inducible by IFNγ in inflammatory hepatitis to promote inflammation in a RIPK3-independent fashion,^41^ MLKL may be induced by the inflammatory response in the COVID-19 lungs.

We also examined the evidence for apoptosis using cleaved-caspase-3 (c-casp3) as a biomarker. C-casp3 was found in the ciliated epithelial cells in the COVID-19 lung, but not in the control lung (Fig. 1c). These data suggest that SARS-CoV-2 infection can induce apoptosis in the ciliated epithelial cells of the COVID-19 lung. C-casp3 was also found in a small number of macrophages in the control lung, suggesting that macrophages may undergo apoptosis under normal conditions (Supplementary information Fig. S4d). Substantially more c-casp3^+^ macrophages were found in the COVID-19 lung relative to control, which is consistent with the accumulation of macrophages in the severe COVID-19 lungs.^30,42^ Thus, apoptosis may mediate the death of epithelial cells and macrophages in the lungs of patients with severe COVID-19.

### Inhibition of RIPK1 kinase reduces the viral load and inflammation in lung organoids infected with SARS-CoV-2

**Fig. 2:**
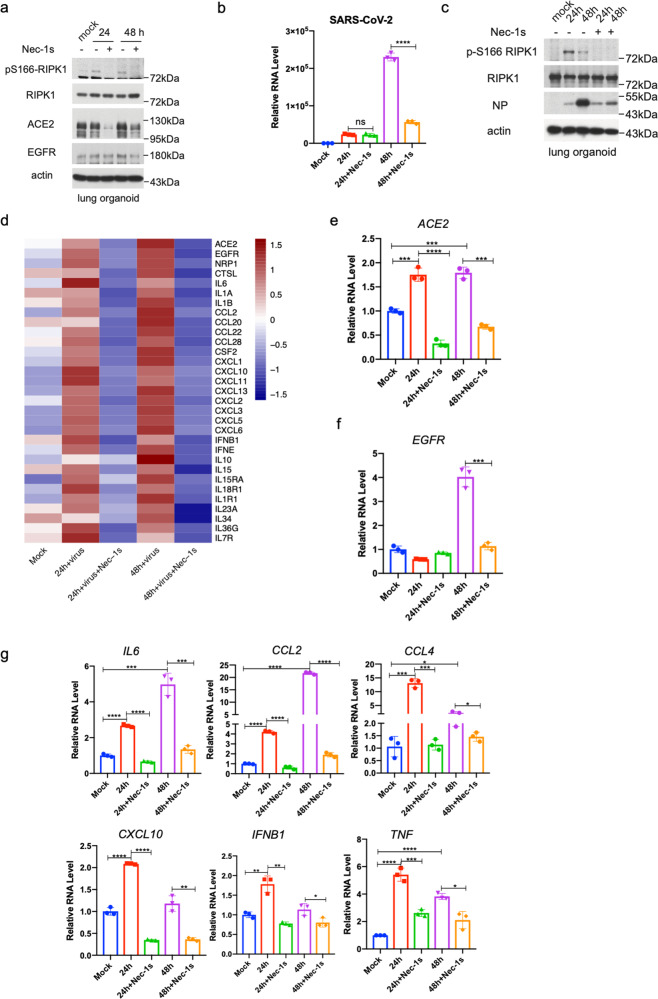
Treatment with Nec-1s reduces SARS-CoV-2-induced inflammation and viral load. **a**–**c** Treatment with Nec-1s inhibits SARS-CoV-2-induced inflammation in the human lung organoids. Human lung organoids were first infected with SARS-CoV-2 at MOI of 1 for 2 h, washed with PBS and replaced with fresh media with 10 μM Nec-1s or vehicle, and incubated for 24 or 48 h. Activated RIPK1 in the lung organoids was determined by western blotting using anti-p-S166 RIPK1 and anti-RIPK1 antibodies. Viral RNA was quantified by RT-qPCR (**b**), and western blotting for NP (**c**). **d**–**f** The lung organoids were treated as in **a** and total RNA was extracted for RNA-seq. The heatmap shows inflammation-related genes that were significantly downregulated after the treatment with Nec-1s (adjusted *P* value < 0.05) (**d**). The levels of ACE2 in the lung organoids were determined by RT-qPCR (**e**) or western blotting (**a**). Actin was used as a loading control. The levels of EGFR in the lung organoids were determined by RT-qPCR (**f**) or western blotting (**a**). **g** The levels of indicated proinflammatory cytokines in the lung organoids treated as in **a** were measured by RT-qPCR. Paired *t*-test was used in RT-qPCR analysis (*P < 0.05, **P < 0.01, ***P < 0.001, ****P < 0.0001).

We next examined the role of RIPK1 in mediating inflammation upon infection of lung organoid culture by SARS-CoV-2. Lower respiratory tract epithelial cultures were established using bronchiolar and the alveolar tissue from patient biopsies.^37^ The obtained tissue was processed for epithelial progenitor cell expansion, followed by differentiation on an air–liquid interface to form two-dimensional (2D) lung organoids composed of the major cell types of the lower respiratory tract. The superficial layers of the lung organoids are ciliated epithelial cells (Supplementary information Fig. S5a). To examine the role of RIPK1 in mediating inflammation induced by SARS-CoV-2, we infected lung organoids with SARS-CoV-2 in the presence or absence of RIPK1 inhibitor Nec-1s.^29,43^ Nec-1s is a further structurally optimized derivative of RIPK1 inhibitor Nec-1^44^ and demonstrates therapeutic potential in a variety of animal models of human inflammatory diseases.^39^ We found that the infection of lung organoids by SARS-CoV-2 promoted the activation of RIPK1 as indicated by p-S166 RIPK1 in the infected lung organoids which was blocked by Nec-1s (Fig. 2a). Furthermore, treatment with Nec-1s in the infected lung organoids reduced the viral load as indicated by the reduction in the coronaviral RNA and NP protein levels (Fig. 2b, c). We additionally tested the effect of 4 different small-molecule RIPK1 inhibitors in SARS-CoV-2-infected human lung organoids and found that all of them inhibited the viral-mediated activation of RIPK1 as indicated by diminished p-S166 RIPK1 and reduced the viral load of SARS-CoV-2 as indicated by the reduction in the levels of viral NP (Supplementary information Fig. S5b).

We next determined the effect of Nec-1s on the transcriptome of human lung organoids infected by SARS-CoV-2. RNA-seq analysis of the lung organoids infected with SARS-CoV-2 in the presence of Nec-1s revealed a considerable reduction in the expression of coronaviral receptor ACE2 relative to SARS-CoV-2-infected organoids cultured in the absence of Nec-1s (Fig. 2d and Supplementary information Fig. S5c). The reduction of ACE2 in SARS-CoV-2-infected lung organoids treated with Nec-1s was confirmed by RT-qPCR at RNA level and western blotting at protein level (Fig. 2a, e). On the other hand, treatment with Nec-1s had no effect on the viral load of SARS-CoV-2 in HeLa cells stably expressing ACE2 (HeLa-ACE2 cells), supporting the importance of the regulation of ACE2 expression by RIPK1 in the control of SARS-CoV-2 replication (Supplementary information Fig. S5d). Treatment with Nec-1s also had no effect on viral load in Huh7 cells, which constitutively express ACE2; and the infection of Huh7 cells by SARS-CoV-2 reduced the expression levels of ACE2, rather than increasing it as in the lung organoids (Supplementary information Fig. S5e). Taken together, these results suggest that RIPK1 does not directly regulate the replication machinery of SARS-CoV-2 and may act by controlling the expression of cellular viral receptor(s), such as ACE2, which is induced in the patient lungs by SARS-CoV-2 which may be important for viral propagation into distal lungs.^37^

EGFR is also known to serve as a host factor in facilitating viral entry for different types of viruses.^45–47^ Interaction of Toll-like receptors with EGFR in airway epithelial cells is known to promote innate immune responses.^48^ A recent study of phosphoproteomics of SARS-CoV-2-infected cells revealed that virus infection leads to the activation of growth factor receptor signaling, including EGFR.^49^ EGFR has been found in a computational modeling study as a potential interacting partner with Spike protein of SARS-CoV-2.^50^ In the RNA-seq analysis of lung organoids, we found that the expression of *EGFR* was upregulated in lung organoids infected with SARS-CoV-2, which was reduced in infected lung organoids treated with Nec-1s (Fig. 2d). Therefore, we sought to confirm the effect of SARS-CoV-2 infection on *EGFR* expression in the lung organoids. We found that EGFR was upregulated at both mRNA and protein levels in the lung organoids infected by SARS-CoV-2, but the expression was reduced by the treatment with Nec-1s (Fig. 2a, f). Downregulation of EGFR by Nec-1s in the lung organoids infected by SARS-CoV-2 may regulate an early immune response against the infection.

Inhibition of RIPK1 by treatment with Nec-1s substantially attenuated the upregulation of many proinflammatory cytokines, including *IL6, CCL2, CCL4, TNF, IL1β, CXCL10, IFNβ*, etc., in the human lung organoids infected by SARS-CoV-2 (Fig. 2d, g). Gene Ontology (GO) analysis of SARS-CoV-2-infected lung organoids treated with Nec-1s or vehicle demonstrated a significant reduction in the expression of viral-induced cytokines and genes associated with NOD-like/Toll-like receptor signaling pathways upon inhibition of RIPK1 (Supplementary information Fig. S5f). Thus, inhibition of RIPK1 reduced the proinflammatory responses activated by SARS-CoV-2 in the human lung organoids. In contrast, SARS-CoV-2 infection had no strong effect on the expression of proinflammatory cytokines in Huh7 cells or HeLa-ACE2 cells (Supplementary information Fig. S5g). Taken together, these results suggest the importance of inflammatory mechanism, which includes the induction of viral receptors, such as ACE2 and EGFR, as well as that of proinflammatory cytokines, activated by RIPK1 in promoting viral replication in human lung organoids.

### Coronaviral RdRp NSP12 interacts with RIPK1 to promote its activation

**Fig. 3:**
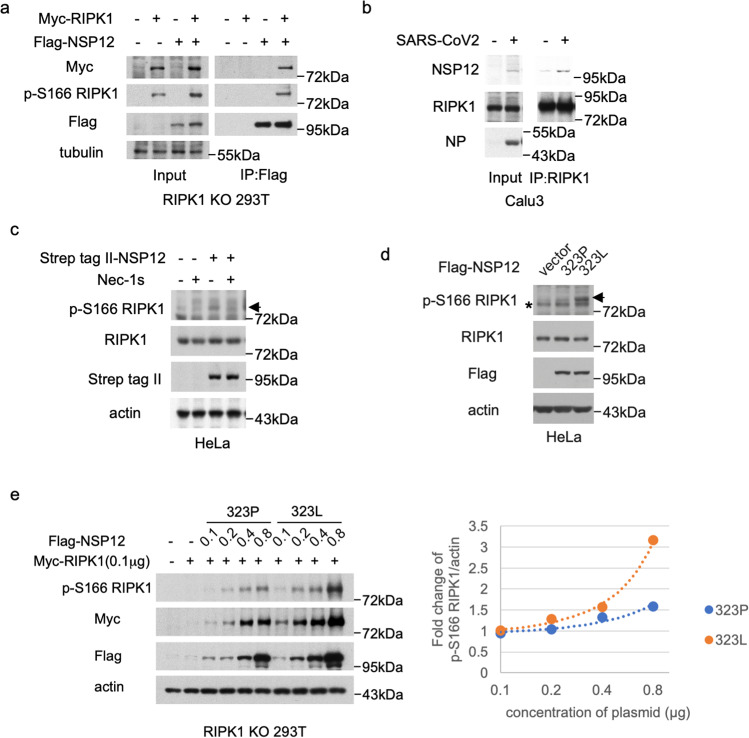
Binding of NSP12 with RIPK1 promotes the activation of RIPK1. **a** RIPK1 KO 293T cells were co-transfected with Myc-RIPK1 and Flag-NSP12 expression vectors as indicated for 20 h. The cell lysates in 1% NP40 lysis buffer were analyzed by immunoprecipitation using anti-Flag M2-affinity gel and followed by western blotting using indicated antibodies. **b** Calu3 cells were infected with SARS-CoV-2 at MOI of 1 for 36 h. The cell lysates in 1% NP40 lysis buffer were analyzed by immunoprecipitation using anti-RIPK1 antibody and followed by western blotting using indicated antibodies. **c** HeLa cells were transfected with Strep tag II-NSP12 expression vector in the absence or presence of Nec-1s for 24 h. The cell lysates were analyzed by western blotting using indicated antibodies. The arrow points to the band of p-S166 RIPK1. **d** HeLa cells were transfected with vector control or expression vectors of NSP12 variants (323P or 323L). The cell lysates were analyzed by western blotting using p-S166 RIPK1 antibody for detection of activated RIPK1 kinase. Actin served as a loading control. The arrow points to the band of p-S166 RIPK1. **e** RIPK1 KO 293T cells were co-transfected with 0.1 μg of Myc-RIPK1 expression vector and different amounts of Flag-NSP12-323P and Flag-NSP12-323L expression vectors as indicated. The cells were lysed with 2× Laemmli sample buffer and analyzed by western blotting using indicated antibodies as shown on the left. Densitometric analysis for the levels of RIPK1 activation (p-S166 RIPK1)/actin is shown on the right. Fold changes were calculated compared to expression of Myc-RIPK1 alone.

We next investigated the mechanism by which SARS-CoV-2 promotes the activation of RIPK1. Inhibition of caspases is commonly required to promote the activation of RIPK1 as the activation of caspase-8 can cleave RIPK1 after the N-terminal kinase domain which in turn inhibits the activation of RIPK1 kinase.^51^ Thus, it was surprising and unusual that infection with SARS-CoV-2 alone was sufficient to promote the activation of RIPK1. We hypothesized that the activation of RIPK1 in SARS-CoV-2-infected cells might be directly mediated by a component encoded by the SARS-CoV-2 genome. The RNA-dependent RNA polymerase (RdRp) (NSP12) in SARS-CoV-2, encoded by the gene *nsp12*, is the central component of coronaviral replication and transcription machinery and is highly conserved in human coronaviruses, including SARS-CoV and MERS-CoV.^52^ RIPK1 was identified as one of the host proteins that can interact with NSP12 protein in a mass spectrometry-based protein–protein interaction study.^53^ We therefore characterized the interaction of NSP12 with RIPK1. We confirmed that NSP12 interacted with RIPK1 (Fig. 3a). Subsequently, the interaction of endogenous NSP12 and RIPK1 was also found in Calu3 cells upon SARS-CoV-2 infection (Fig. 3b). Furthermore, we found that the levels of activated RIPK1 were higher in 293T cells co-transfected with NSP12 and RIPK1 expression vectors than that of cells transfected with the RIPK1 expression vector alone (Fig. 3a). The expression of NSP12 also promoted the activation of endogenous RIPK1 in HeLa cells (Fig. 3c).

The structure of NSP12 in SARS-CoV-2 contains a C-terminal RdRp domain (amino acid residues S367 to F920), which carries the RNA polymerase catalytic activity common to all RNA viruses and an N-terminal nidovirus RdRp-associated nucleotidyltransferase (NiRAN) extension (amino acid residues D60 to R249) unique to Nidoviruses.^52^ The RdRp domain and NiRAN extension are connected by an interface domain (amino acid residues A250 to R365). We found that the interface domain and RdRp domain of NSP12 could interact with RIPK1, and the kinase domain of RIPK1 was required for the binding with NSP12 (Supplementary information Fig. S6a, b).

Pro323 in the interface domain of NSP12 is located in a short, twisted α-helix with its side chain exposed on the surface of the hydrophobic cavity at the backside of the polymerase complex.^52^ Exchanging a proline residue with the hydrophobic leucine in NSP12 increases the hydrophobic character of the cavity and is predicted to lead to structural changes.^54^ Because the interface domain of NSP12 is involved in interacting with RIPK1, we compared the ability of NSP12 323P and NSP12 323L in activating RIPK1. We found that the expression of NSP12 323L induced greater activation of RIPK1 compared to that of NSP12 323P (Fig. 3d). NSP12 323L demonstrated increased binding and stronger activation of RIPK1 over a range of concentrations compared to that of NSP12 323P (Fig. 3e and Supplementary information Fig. S6c).

**Fig. 4:**
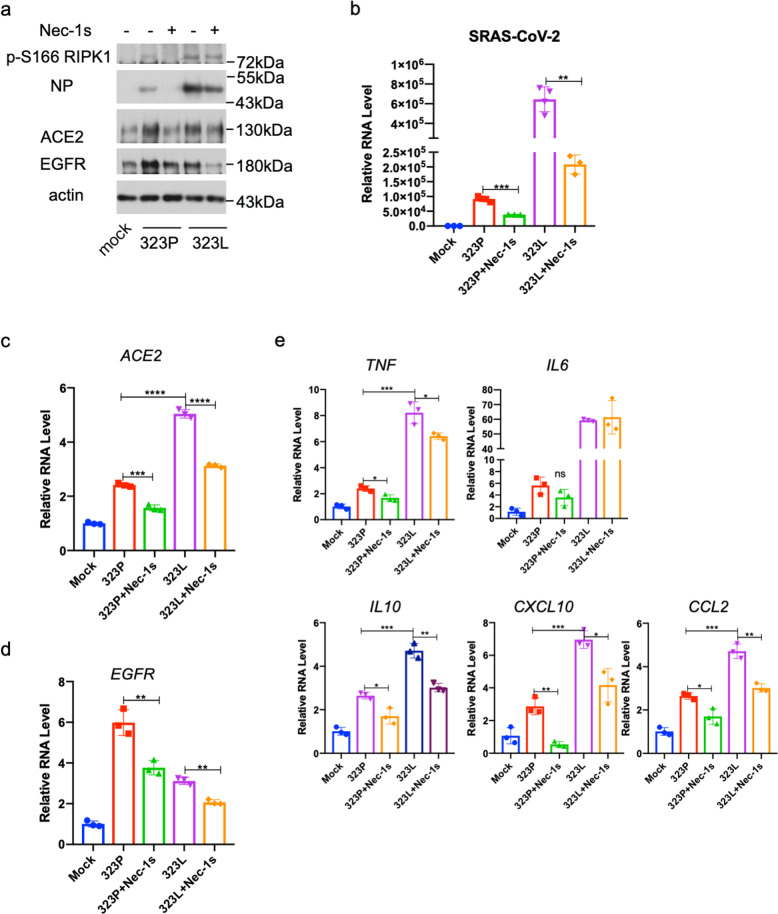
Nec-1s inhibits propagation of 323P and 323L SARS-CoV-2 and inflammation. **a**–**e** The human lung organoids were infected with 323P SARS-CoV-2 or 323L SARS-CoV-2 for 2 h, washed with PBS and replaced with fresh media with 10 μM Nec-1s or vehicle, and incubated for additional 48 h. The cells were lysed with 2× Laemmli sample buffer and analyzed by western blotting using indicated antibodies (**a**). Total RNA was extracted; viral titers (**b**) and the expression of ACE2 (**c**), EGFR (**d**) and cytokines (**e**) were quantified by RT-qPCR. Paired *t*-test was used in qPCR analysis (*P < 0.05, **P < 0.01, ***P < 0.001).

To determine the functional significance of the differential interaction mediated by the original variant NSP12 323P and NSP12 323L encoded by the C14408T variant with RIPK1, we isolated a virus strain carrying the C14408T (NSP12 P323L) mutation from a patient nasopharyngeal swab. Compared to the 323P variant, the 323L variant was significantly more infectious and promoted stronger RIPK1 activation in human lung organoids (Fig. 4a, b). Interestingly, treatment with RIPK1 inhibitor Nec-1s was able to inhibit RIPK1 activation and reduce the level of viral replication as indicated by NP expression in the human lung organoids infected by either the 323P or 323L variants (Fig. 4a, b). Treatment with Nec-1s reduced the levels of ACE2 and EGFR in the lung organoids infected with 323L variant as that of 323P variant (Fig. 4c, d). Finally, treatment with Nec-1s also downregulated the mRNA levels of multiple proinflammatory cytokines in the lung organoids induced by both 323P and 323L virus (Fig. 4e). Thus, inhibition of RIPK1 may be able to reduce the replication of different SARS-CoV-2 variants as well as mitigate the inflammatory response induced by SARS-CoV-2. These results suggest that the increased capacity of NSP12 323L to bind and activate RIPK1 may act as a mechanism to promote the propagation of SARS-CoV-2 which may be translated into increased infectivity in humans.

### Inhibition of RIPK1 reduces SARS-CoV-2 viral loads in vivo

**Fig. 5:**
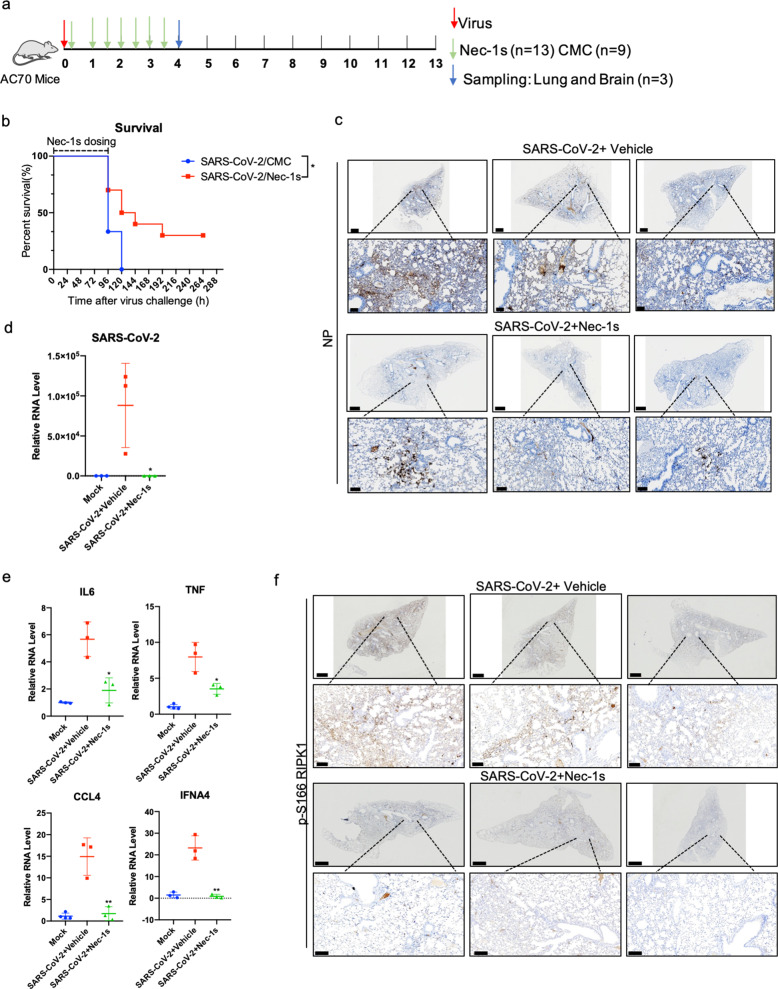
RIPK1 inhibitor protects CAG-hACE2 transgenic mice (AC70) infected by SARS-CoV-2. **a** Experimental design of in vivo study. AC70 transgenic mice (6–8 weeks of age, male) were intranasally infected with 20 μL 1 × 10^4^ TCID_50_ SARS-CoV-2. Two hours after infection, the mice were intragastrically administered with Nec-1s (*n* = 13) (50 mg/kg, dissolved in 0.5% Carboxymethyl Cellulose (CMC)) or 0.5% CMC vehicle alone (*n* = 9), and the dosing was repeated every 12 h. On the day 4 post-infection, 3 mice in each group were sacrificed and their lungs and brains were dissected. The remaining 10 mice in the treatment group (Nec-1s treated for 4 days) and 6 mice in the vehicle group stopped dosing after day 4 and were used to observe the survival rate. **b** Survival of AC70 transgenic mice after intranasal SARS-CoV-2 infection with intragastric administration of Nec-1s or vehicle. The statistical significance of the survival curve was estimated according to the method of Kaplan and Meier, and the curve was compared with the generalized Wilcoxon test (*P < 0.05). **c**, **d** Treatment with Nec-1s inhibits SARS-CoV-2 infection in AC70 transgenic mice. The lungs of mice in the control group and Nec-1s-treated group were fixed and stained for SARS-CoV-2 NP by IHC (**c**). Scale bars, 900 μm. The lower row shows the enlarged images of specified areas above. Scale bars, 100 μm. Freshly isolated mouse lungs were extracted with Trizol for isolating RNA. After reverse transcription, the virus RNA was analyzed by RT-qPCR (**d**). Paired *t*-test was used in RT-qPCR analysis (*P < 0.05). **e** Treatment with Nec-1s inhibits inflammation in the lungs of AC70 transgenic mice infected with SARS-CoV-2. The lungs of mice in the control group (*n* = 3) and Nec-1s-treated group (*n* = 3) were extracted with Trizol, and the expression of cytokines was analyzed by RT-qPCR. Paired *t*-test was used in RT-qPCR analysis (*P < 0.05, **P < 0.01). **f** RIPK1 was activated in the lungs of AC70 transgenic mice with SARS-CoV-2 infection. IHC staining of p-S166 RIPK1 in the lungs of control or Nec-1s-administrated AC70 transgenic mice infected with SARS-CoV-2 was shown. Scale bars, 900 μm. The lower row shows the enlarged images of specified areas above. Scale bars, 100 μm.

AC70 transgenic mice expressing high levels of hACE2 driven by the ubiquitous CAG promoter in different organs are highly susceptible to SARS-CoV infection.^55^ In response to nasal infection by SARS-CoV, these transgenic mice experience severe clinical manifestations, which leads to 100% mortality. High viral titers were detected in the lungs of AC70 transgenic mice after infection associated with induction of inflammatory mediators and thus, it is considered to be a model of severe SARS-CoV.^55^ We found that similar to that of SARS-CoV infection, AC70 transgenic mice showed 100% mortality 5 days after intranasal infection of SARS-CoV-2; and from this we conclude that they are also highly susceptible to SARS-CoV-2 and provide a model for severe COVID-19. Therefore, we tested the in vivo therapeutic efficacy of RIPK1 inhibitor Nec-1s in AC70 transgenic mice after infected by SARS-CoV-2 323P variant. Two hours after intranasal infection of SARS-CoV-2, Nec-1s was administered intragastrically at a dose of 50 mg/kg, once every 12 h for 4 days (Fig. 5a). Administration of Nec-1s for only 4 days provided statistically significant improvement on the survival of SARS-CoV-2-infected mice, from 0% to 50% on day 5 and 30% of the mice recovered completely (Fig. 5b and Supplementary information Videos S1 and S2). Since AC70 mice died quickly after the onset of SARS-CoV-2 infection, their weight loss during the infection was not obvious; however, Nec-1s dosing allowed rapid increase of their body weights after day 7 (Supplementary information Fig. S7a). Histopathological analysis showed reduction in virus-induced segmental consolidation of the lungs in Nec-1s-treated mice (Supplementary information Fig. S7b). Both NP immunohistochemical staining and RT-qPCR showed that treatment with Nec-1s also greatly reduced the SARS-CoV-2 viral load in lung (Fig. 5c, d). The reduction of lung viral load in Nec-1s-administrated AC70 mice infected by SARS-CoV-2 was further confirmed by viral plaque-forming unit assay (Supplementary information Fig. S7c, d). SARS-CoV-2 infection of AC70 transgenic mice upregulated the expression of multiple inflammatory cytokines in the lungs, such as TNF, IL6, CCL4 and IFNA4, which were downregulated by the treatment with Nec-1s (Fig. 5e). Consistent with the activation of RIPK1 by SARS-CoV-2 infection in the lungs of COVID-19 patients, activated RIPK1 was also found in the lungs of SARS-CoV-2-infected AC70 transgenic mice and was reduced in the lungs of the mice treated with Nec-1s (Fig. 5f).

**Fig. 6:**
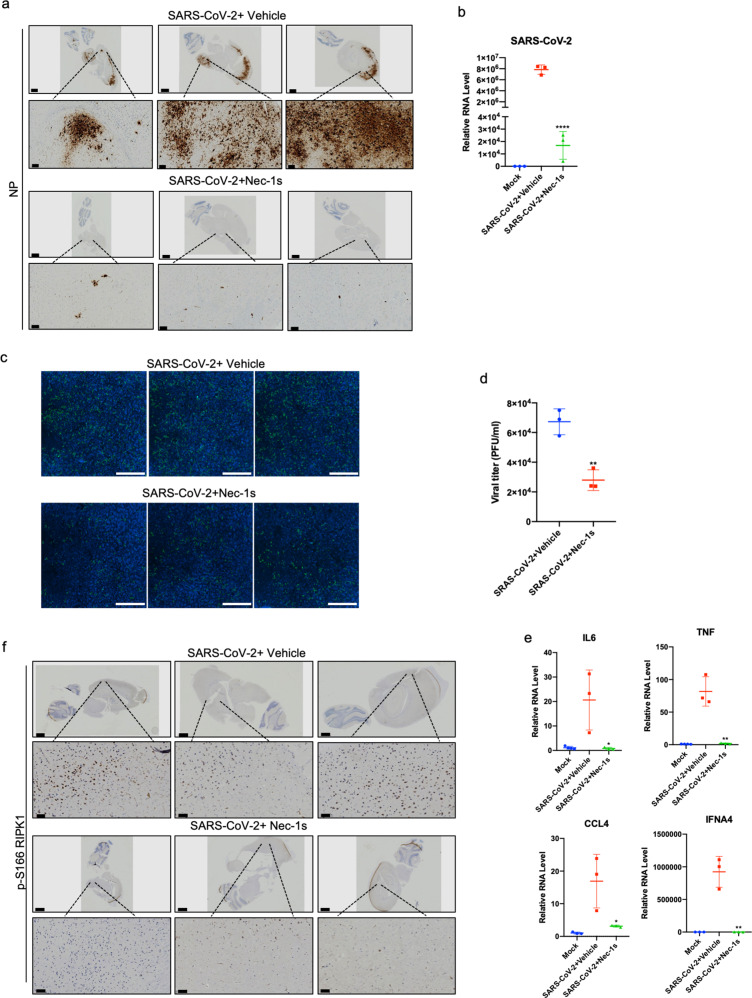
Nec-1s treatment reduces the viral loads in the brain of AC70 transgenic mice. **a** IHC staining with antibody against viral NP on the saggital whole brain sections of the SARS-CoV-2-infected AC70 transgenic mice with or without Nec-1s treatments. Scale bars, 1000 μm. The lower row shows the enlarged images of specified areas above. Scale bars, 100 μm. Viral NP was robustly detected in multiple brain regions, including the olfactory bulb, the deep cortical layers, the connected hippocampal subiculum, the hindbrain/medulla and the cerebellar dentate nucleus. **b** Freshly isolated mouse brains were extracted with Trizol for isolating RNA. After reverse transcription, the virus RNA was analyzed by RT-qPCR. Paired *t*-test was used in RT-qPCR analysis (****P < 0.0001). **c**, **d** The brain tissues of viral-infected AC70 mice with or without Nec-1s treatment (1 g) were cryogenically ground in 1 mL DMEM medium. After fully grinding and centrifugation, the supernatant was collected and stored at –80 °C. 4 × 10^4^ Vero-E6 cells were seeded in 96-well plates for 23 h. The supernatant (50 μL) was added to the cells and incubated for 24 h. The SARS-CoV-2 infection was detected by immunofluorescence using COVID-19 convalescent sera (**c**). Scale bars, 1000 μm. Infectious clones are automatically quantified by Cytation 5 (**d**). Paired *t*-test was used in viral titer analysis (**P < 0.01). **e** The brains of mice in the control group (*n* = 3) and Nec-1s-treated group (*n* = 3) were extracted with Trizol for total RNA, and the expression of cytokines was analyzed by RT-qPCR. Paired *t*-test was used in RT-qPCR analysis (*P < 0.05, **P < 0.01). **f** RIPK1 was activated in the brains of AC70 transgenic mice with SARS-CoV-2 infection. IHC staining of p-S166 RIPK1 in the brains of control or Nec-1s-administrated AC70 transgenic mice infected with SARS-CoV-2 was shown. Scale bars, 1000 μm. The lower row shows the enlarged images of specified areas above. Scale bars, 50 μm.

SARS-CoV-2 has been reported to infect the brain of COVID-19 patients as well as experimental mouse models.^56–58^ Therefore, we next analyzed the viral titers and the expression of cytokines in the brains of SARS-CoV-2-infected AC70 transgenic mice with or without Nec-1s administration. Surprisingly, we found significant viral invasion in the brains of infected AC70 mice. Histopathology and immunohistochemical analysis of SARS-CoV-2 NP in the virus-infected mouse brains showed massively infected neurons as well as glial cells. The viral infection can be found from the olfactory bulb, spread to the deep cortical layers, and the connected hippocampal subiculum. In addition, the hindbrain/medulla and cerebellar dentate nucleus also showed robust NP staining. Such staining pattern suggests SARS-CoV-2 virus spread inside the brain through at least one synaptic connection (Fig. 6a).

Interestingly, the treatment with Nec-1s greatly reduced the SARS-CoV-2 viral load in the brains (Fig. 6a–d). Corresponding to the heavy viral loads, activated RIPK1 and highly elevated expression of proinflammatory cytokines were observed in the brains of infected mice. Nec-1s treatment can effectively inhibit the activation of RIPK1 and reduce the expression of proinflammatory cytokines in the brain (Fig. 6e, f). In contrast, we failed to uncover the evidence of apoptosis and necroptosis in the CNS, which might be due to the acute nature of this model. Thus, virus-mediated activation of RIPK1 may be primarily involved in promoting the production of proinflammatory cytokines in this model. Taken together, these data suggest the therapeutic potential of RIPK1 inhibitors for the treatment of severe COVID-19 — not only for inhibiting inflammatory cytokine storm, but also for reducing viral loads.

## Discussion

The ongoing COVID-19 pandemic is exerting an unprecedented devastating effect on public health, the worldwide economy and modern life style. This is largely due to the ability of SARS-CoV-2 to promote the development of severe COVID-19 in a substantial subset of infected population. To mitigate this ongoing public health disaster, it is crucial for us to understand the relationship of SARS-CoV-2 and the innate immune response which is normally a part of host defense response against pathogens. We demonstrate the ability of SARS-CoV-2 in promoting the activation of RIPK1 and the unexpected role of RIPK1-mediated innate immunity in promoting viral propagation and CNS invasion by SARS-CoV-2. The ability of RIPK1 inhibition to block SARS-CoV-2 invasion to the CNS suggests that the virus-mediated RIPK1 activation may play an important role in breaking the blood-brain-barrier (BBB) which normally protects the CNS. We have recently reported the role of necroptosis in cerebromicrovascular endothelial cells mediated by RIPK1 and RIPK3 in promoting the BBB damage in Alzheimer’s disease^59^ and in Nek1-deficient mice.^60^ Future studies may be needed to directly investigate the role of necroptosis in mediating CNS invasion by SARS-CoV-2.

Unexpectedly, we found that the RdRp of SARS-CoV-2 could promote the activation of RIPK1 to facilitate viral replication. However, since inhibition of RIPK1 by Nec-1s did not reduce viral loads in HeLa cells stably expressing ACE2 or in Huh7 cells which show a reduction of ACE2 upon viral infection, Nec-1s is not likely acting as a direct inhibitor of the RdRp replication machinery. Thus, the interaction of RIPK1 with NSP12 likely happens outside of the RdRp complex. Activation of RIPK1 by NSP12 stimulates the expression of viral receptors, such as ACE2 and EGFR, which in turn promotes the viral replication. Thus, SARS-CoV-2 may be able to hijack a part of the RIPK1-mediated innate immune response to facilitate its propagation under certain conditions. In cells with a high viral load, the NSP12 RdRp can interact with RIPK1 to promote its activation. Activation of RIPK1 can stimulate the expression of ACE2, EGFR and inflammatory cytokines in viral-infected cells which collectively is likely to be important in promoting SARS-CoV-2 replication. Furthermore, the increased ability of SARS-CoV-2 323L variant to stimulate RIPK1 activation and replicate in the lung organoids also suggests that the NSP12 P323L mutation may contribute to the spread of this SARS-CoV-2 variant around the world. In addition, since the structural changes in NSP12 introduced by P323L mutation may contribute to the resistance to remesdesivir,^54,61^ RIPK1 inhibition may offer a therapeutic strategy to reduce viral propagation.

We show that SARS-CoV-2 infection can stimulate the expression of ACE2, EGFR and proinflammatory cytokines in a RIPK1-dependent manner. Elevated levels of proinflammatory cytokines may in turn provide a positive feed-forward mechanism to further stimulate the expression of ACE2. However, because inhibition of RIPK1 by Nec-1s can also increase the survival of ACE2 transgenic mice infected by SARS-CoV-2, the activation of RIPK1 may be able to promote viral replication through multiple mechanisms that include facilitating the expression of ACE2 as well as modulating RIPK1-mediated innate immune responses involving EGFR and the production of proinflammatory cytokines.

Cytokine storm, characterized by a systemic inflammatory reaction with increased production of proinflammatory cytokines such as IL6, TNF and inflammatory chemokines such as CCL2, CCL3 and CXCL10, has been proposed to play a key role in mediating mortality in severe COVID-19.^25,62–64^ Since RIPK1 kinase activity has an established role in mediating systemic inflammatory response,^14^ the activation of systemic inflammatory response in severe COVID-19 can serve as a biomarker for RIPK1 activation. Inhibition of RIPK1 by either genetic or pharmacological means in animal models is highly effective in blocking TNF-induced sepsis, including attenuation of hypothermia and complete rescue of lethality.^65,66^ Multiple RIPK1 inhibitors have been advanced beyond Phase I safety studies in human clinical trials for the treatment of human inflammatory and neurodegenerative diseases, including rheumatoid arthritis, psoriasis, inflammatory bowel disease, Alzheimer’s disease and amyotrophic lateral sclerosis.^14^

Our results described above demonstrate that NSP12, the central component of coronaviral replication and transcription machinery of SARS-CoV-2, is a multi-functional protein that is not only directly involved in the replication of viral RNA as known previously, but can also promote the activation of RIPK1 which may in turn promote inflammation and the expression of host viral receptor ACE2 and additional factors such as EGFR which can enhance viral survival and propagation. The role of RIPK1 in driving inflammatory response in human diseases has been well established.^14^ We conclude that SARS-CoV-2 is able to hijack the host inflammatory response mediated by RIPK1 to its own advantage to enhance viral survival and replication in host cells. Since inhibition of RIPK1 can reduce the replication of both 323P and 323L SARS-CoV-2 in human lung organoids, our results suggest that RIPK1 kinase inhibitors may provide an effective therapy for severe COVID-19 as well as reducing the spread of SARS-CoV-2 variants. Our data support further clinical studies of a RIPK1 inhibitor for the treatment of severe COVID-19.

## Materials and methods

### Ethics statement

This study was reviewed and approved by the Medical Ethical Committee of Shenzhen Third People’s Hospital (2020-231). The bronchoalveolar lavage fluid (BALF), lung and peripheral blood mononuclear cells (PBMCs) and postmortem lung samples were obtained from the collection of the Shenzhen Third People’s Hospital (one COVID-19 patient). Additional postmortem lung samples were from the collection of Wuhan Tongji Medical College (three COVID-19 patients). The nasopharyngeal swab from patients was used to isolate WT and C14408T variant virus with signed permission. The disease severity was defined to be moderate, severe and critical status, according to the ‘Diagnosis and Treatment Protocol of COVID-19 (the 7th Tentative Version)’ by National Health Commission of China issued on March 3, 2020 (http://www.nhc.gov.cn/yzygj/s7653p/202003/46c9294a7dfe4cef80dc7f5912eb1989.shtml). All control lung tissues were from normal tissues far from the tumor of lung adenocarcinoma patients.

### Cells and plasmids

HeLa and HEK-293T cells were cultured in Dulbecco’s Modified Eagle Medium (DMEM, Gibco). Strep tag II-NSP12 was kindly provided by Professor Nevan J. Krogan (University of California San Francisco). Flag-NSP12 was subcloned from Strep tag II-NSP12 into pcDNA 3.1 vector. Flag-NSP12 P323L was generated using MutExpress II mutagenesis kit (Vazyme Biotech).

### Human lung organoid culture

Lower respiratory tract epithelial cultures were derived from patient biopsy residues similar to published airway organoids derived from epithelial progenitor cells.^37^ Lung tissue was surgically excised from the tissue residue of a lung donor for the healthy part of the lung parenchyma. The obtained tissue was then washed with sterile PBS, dissected for the healthy epithelium portion of the tissue, and cut into small pieces using sterile scissors. The tissue pieces were then enzyme-digested by Dispase I (Stemcell Technologie, CA), centrifuged to purify the cell portion, plated in culture dishes with 3T3 fibroblast cells as feeder cells, and expanded using PneumaCult^TM^-Ex Plus Medium (Stemcell Technologie, CA). After progenitor cell expansion, the cultured cells were transferred onto 0.4 μm transwells on a 24-well plate, and differentiated on an ALI culture using PneumaCult^TM^-ALI Medium (Stemcell Technologie, CA). All the human sample-related work was approved by the Medical Ethical Committee of Shenzhen Third People’s Hospital (2020-231) and the ethics committee of Shenzhen Institutes of Advanced Technology, Chinese Academy of Sciences (SIAT-IRB-200215-H0415). Written informed consents were obtained from all participating patients.

### SARS-CoV-2 preparation and infection

All studies involving SARS-CoV-2 infection were conducted in the biosafety level-3 (BLS-3) laboratory of Shenzhen Third People’s Hospital. SARS-CoV-2 isolate SZTH-003 (323P) and SARS-CoV-2 isolate SZ454 (323L) were sourced from COVID-19 patients. SARS-CoV-2 was amplified once in Vero-E6 cells and viral stocks were stored at –80 °C. Human lung organoids were incubated with SARS-CoV-2 at 1 MOI for 2 h at 37 °C. Subsequently, infection medium was removed and cells were incubated in normal media with or without 10 μM Nec-1s in 37 °C, 5% CO_2_ condition and harvested for analysis at indicated time points. Additional four RIPK1 inhibitors have been tested for anti-SARS-CoV-2 ability, including CMP21 (Takeda), GSK2982772 (GlaxoSmithKline), GSK963 (GlaxoSmithKline), and QY10-40, using the same protocol. 0.1% DMSO in culture media was used as vehicle control.

### Evaluating the effect of Nec-1s in CAG-hACE2 transgenic mouse (AC70) model

The animal experiments were conducted in the BLS-3 animal facility approved for the studies of SARS-CoV-2. The experiment protocol has been approved by the Animal Ethics Committee of the Second Military Medical University. AC70 CAG-hACE2 transgenic mice (Shanghai Model Organisms, NM-TG-200002) (6–8 weeks of age, male) were intranasally infected with 20 μL 1 × 10^4^ TCID_50_ SARS-CoV-2. Two hours after infection, the mice were intragastrically administered with Nec-1s (50 mg/kg, dissolved in Carboxymethyl Cellulose, CMC) or CMC vehicle alone, and the dosing was repeated every 12 h. On the day 4 post-infection, three mice in each group were sacrificed and their lungs and brains were removed. The lung and brain samples were extracted with Trizol (Invitrogen™, 15596026) for isolating RNA, solubilized in DMEM medium for detection of viral titer, or fixed with formalin for 48 h and prepared for paraffin embedding and subsequent HE and IHC staining.

### Determination of viral titer in mouse tissues

Mouse lung or brain tissue (1 g) was cryogenically ground in 1 mL DMEM medium. After fully grinding and centrifugation, the supernatant was collected and stored at –80 °C. In all, 4 × 10^4^ Vero-E6 cells were seeded in 96-well plates for 12 h and 50 μL supernatant was added to cells. After 24 h of virus incubation, the SARS-CoV-2 infection was detected by immunofluorescence using COVID-19 convalescent sera. Infectious clones were automatically quantified by Cytation 5.

### RT-qPCR

Total RNA was extracted with TRIzol™ Reagent in accordance with the manufacturer’s instructions and reverse-transcribed into cDNA with a High-Capacity cDNA Reverse Transcription Kit (Takara, RR036A). The expression levels of indicated RNA were determined by RT-qPCR analysis using Power SYBR Green PCR Master Mix (Vazyme, Q311-02). Primers used in RT-qPCR reactions were listed in Supplementary information Table S2.

### Western blotting

Cell lysates were boiled in 2× Laemmli sample buffer containing 10% βME (Sigma, M3148). Cell lysates were analyzed by SDS-PAGE and then transferred onto polyvinylidene difluoride (PVDF) membranes (Millipore, IPFL10100). After blocking with 5% BSA in TBS buffer containing 0.05% Tween 20, the blot was sequentially probed with primary antibodies and the horseradish peroxidase (HRP)-conjugated secondary antibody. Protein bands were detected by SuperSignal West Pico Chemiluminescent substrate (Bio-Rad). The following antibodies were used: anti-ACE2 (Abcam, ab108209), anti-SARS-CoV-2 nucleoprotein (Sino Biological, 40588-T62), anti-p-hRIPK1 (Cell Signaling Technology, 65746), anti-RIPK1 (Cell Signaling Technology, 3493), anti-EGFR (Cell Signaling Technology, 4267), anti-NSP12(Cell Signaling Technology), anti-Flag (Sigma, F1804), anti-Myc (Sigma, C3956), anti-Strep tag II (Abcam, ab76949), anti-Actin (TransGen Biotech, HC201) and anti-Tubulin (TransGen Biotech, HC101).

### IHC staining

Formalin-fixed paraffin-embedded lung tissue was cut into 4 μm sections and mounted on frosted glass slides. After deparaffinization and rehydration, slides were submerged into pH 8.0 EDTA buffer and boiled for 2 min with high pressure for antigenic retrieval. Slides were washed in PBS, incubated in 3% hydrogen peroxide for 10 min and then blocked with 1% bovine serum albumin (BSA) for 1 h. The following primary antibodies were used: anti-p-hRIPK1 (Cell Signaling Technology, 44590), anti-p-mRIPK1 (BioLynx), anti-RIPK1 (BioLynx, Clone 59), anti-p-MLKL (Abcam, ab187091), anti-p-RIPK3 (Cell Signaling Technology, 93654), anti-CD68 (Zymed, San Francisco, CA) and anti-SARS-CoV/SARS-CoV-2 nucleoprotein (Sino Biological, 40143-T62 and MM05), anti-CD66B (Biolegend, 305102), anti-cleaved Caspase-3 (Cell Signaling Technology, 9661). Slides were then washed in PBST (PBS plus 0.1% Tween 20), incubated with the biotinylated secondary antibody (Zymed, San Francisco, CA) and visualized with 3,3′-diaminobenzidine (DAB) under the microscope.

### Immunoprecipitation

Cells were collected and washed with PBS, then lysed with 1% NP40 lysis buffer (50 mM Tris, pH 7.4, 150 mM NaCl, 1% NP40, 5 mM β-glycerophosphate, 2.5 mM sodium pyrophosphate, 5 mM NaF, 200 μM Na_3_VO_4_, supplemented with protease inhibitors cocktail) on ice for 30 min. The lysates were centrifuged at 15,000 rpm at 4 °C for 10 min, and the supernatants were transferred to new tubes and incubated with anti-Flag M2-affinity gel (Sigma, A2220). After incubation, beads were washed four times with lysis buffer, and the immunocomplex was eluted from beads by 2× Laemmli buffer.

### RNA-seq data analysis

The data were analyzed according to the method established by Mihaela et al.^67^ Briefly, the raw FASTQs were aligned to human GRCh38 as the reference genome by HISAT2.^68^ FeatureCounts^69^ was used to generate gene count data for each sample. DESeq2^70^ was used to perform differential expression analysis with gene count data. A gene is considered significant with adjusted *P* value < 0.05 and absolute log_2_FC > 2. For differentially expressed genes (DEGs), KEGG pathway analysis was performed with clusterProfiler,^71^ which supports statistical analysis and visualization of functional profiles for genes and gene clusters. The heatmap was generated with pheatmap function in R v3.6.2 using transcripts per million (TPM) calculated with gene count data. The raw count matrix was converted to TPM matrix and then analyzed with PCAtools package in R. Mitochondrial genes were removed and principal component analysis (PCA) was performed with pca function with removeVar = 0.1. PCA plot was generated with biplot function.

### scRNA-seq library construction

The scRNA-seq libraries were prepared with Chromium Single Cell 3’ Reagent Kits v3 (10× Genomics; PN-1000075, PN-1000073 and PN-120262) following the user guide provided using 10× genomic platform. Briefly, Gel beads in Emulsion (GEM) were generated by combining barcoded Gel Beads, a Master Mix containing 20,000 cells, and Partitioning Oil onto Chromium Chip B. Reverse transcription (RT) takes place inside each GEM, after which cDNAs are pooled for amplification and library construction in bulk. Finished library molecules consisted of Illumina adapters and sample indices, allowing pooling and sequencing of multiple libraries on a next-generation short-read sequencer.

### Public healthy control data

The lung scRNA-seq data from healthy controls were acquired from the Gene Expression Omnibus (GEO) database with the series number GSE122960, which contained the data of lung tissues from eight lung transplant donors generated using 3’ V2 chemistry kit on Chromium single cell controller (10× Genomics).^72^ Filtered feature-barcode matrix was used in the following analysis.

### scRNA-seq data alignment and sample aggregating

The Cell Ranger Software Suite (version 3.1.0) was used to perform sample de-multiplexing, barcode processing and single-cell 3’ UMI counting with human GRCh38 as the reference genome. Specifically, splicing-aware aligner STAR was used in FASTQ alignment. Cell barcodes were then determined based on the distribution of UMI count automatically. Following criteria were then applied to each cell of all samples; i.e., gene number between 200 and 6000 (7000 for COVID-19 in the lung tissue/BALF/PBMC integration analysis), UMI count above 1000 and mitochondrial gene percentage below 0.1. Finally, filtered gene-barcode matrix of all samples was integrated with Seurat v3 to remove batch effect across different samples. In parameter settings, the first 50 dimensions of CCA and PCA were used.

### Dimensionality reduction and clustering

The filtered gene-barcode matrix was first normalized using the ‘LogNormalize’ method in Seurat v3 with default parameters. The top 2000 variable genes were then identified using ‘vst’ method in Seurat FindVariableFeatures function. Variables nCount_RNA and percent.mito were regressed out in the scaling step and PCA was performed using the top 2000 variable genes. Then Uniform Manifold Approximation and Projection (UMAP) was then performed on the top 50 principal components for visualizing the cells. Meanwhile, graph-based clustering was performed on the PCA-reduced data for clustering analysis with Seurat v3, with the resolution being set at 1.0 for lung tissue analysis and 1.2 for lung tissue/BALF/PBMC integration analysis.

### Statistics

All statistical analyses are performed using GraphPad Prism software. Paired *t*-test was used in RT-qPCR analysis. Data were derived from the average of three biological replicate experiments, and calculated as the means ± SEM. *P < 0.05, **P < 0.01, ***P < 0.001, ****P < 0.0001.

## Supplementary information

Supplementary Fig. S1

Supplementary Fig. S2

Supplementary Fig. S3

Supplementary Fig. S4

Supplementary Fig. S5

Supplementary Fig. S6

Supplementary Fig. S7

Supplementary Table S1

Supplementary Table S2

Supplementary Video S1

Supplementary Video S2

Supplementary Video legends

## Data Availability

The complete genome of 323L virus in this paper has been deposited in the Genome Warehouse in National Genomics Data Center, Beijing Institute of Genomics, Chinese Academy of Sciences, under accession number(s) WGS018680 (SZ454), which is publicly accessible at https://ngdc.cncb.ac.cn/gwh/. The genomic sequence of 323P virus (SZTH-003) has been deposited in the Global Initiative of Sharing All Influenza Data (GISAID, EPI_ISL_406594). The RNA-seq data in this paper have been deposited in the Genome Sequence Archive for human in National Genomics Data Center, Beijing Institute of Genomics, Chinese Academy of Sciences under the accession number HRA000694, which is publicly accessible at https://ngdc.cncb.ac.cn/gsa-human.

## Code availability

The source code and software pipeline for repeating our analyses are available upon request.
