# Supplementary material for: SARS-CoV-2 promotes RIPK1 activation to facilitate viral propagation
Source: Cell Res. 2021 Oct 18;31(12):1230–43. doi: 10.1038/s41422-021-00578-7 (PMC8522117; doi:10.1038/s41422-021-00578-7)
Supplement: Supplementary file 1 — Supplementary Fig. S1 [file 41422_2021_578_MOESM1_ESM.pdf]

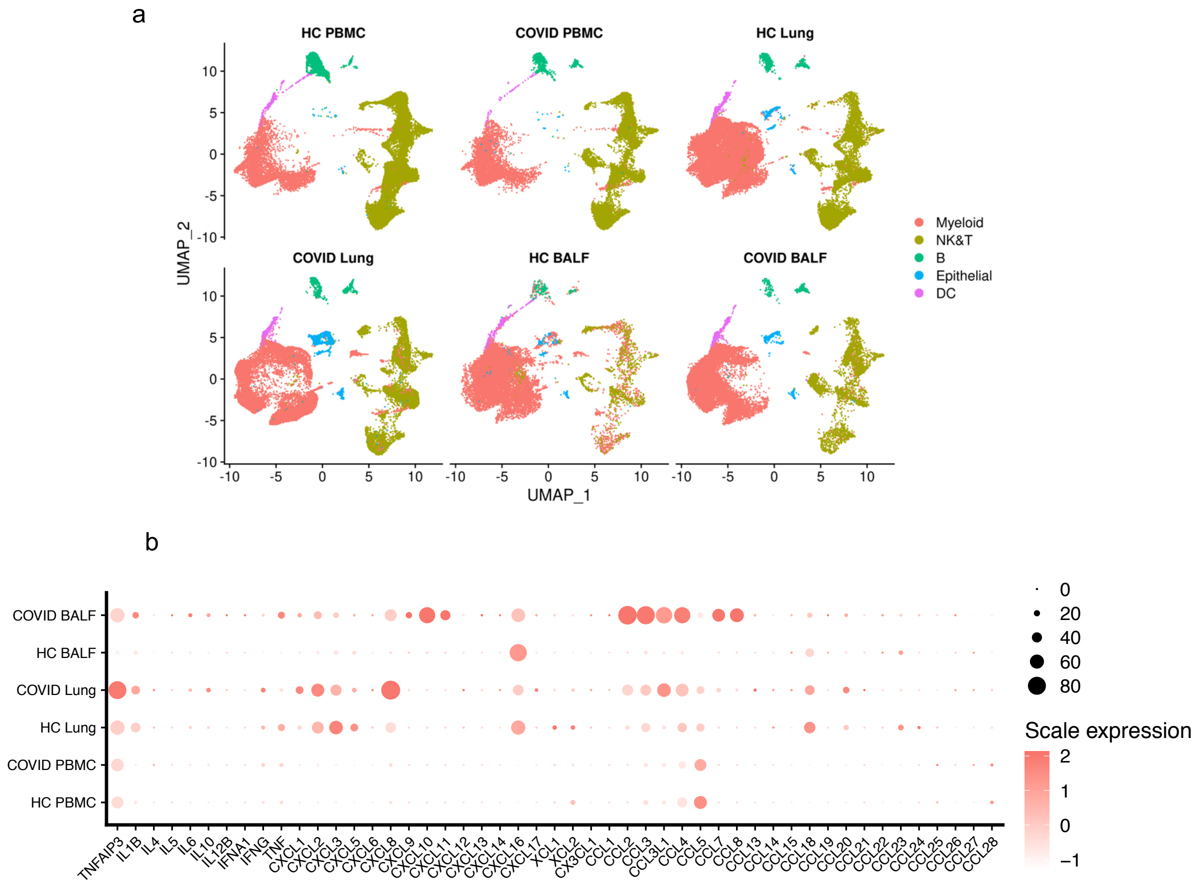

Figure S1 Strong induction of inflammatory response in the lung of severe COVID-19.

a-b. The single-cell sequencing data of 1 PBMC, 3 BALF, and 4 lung tissues from patient 1 and 3 control PBMCs, 3 control BALFs, 3 control lungs were integrated, 143,015 cells were obtained and divided into myeloid cells, NK & T cells, B cells, dendritic cells (DC) and epithelial cells according to specific markers (**a**). The heatmap shows the expression of indicated cytokines and chemokines in the patient and controls (**b**).
