# Supplementary material for: SARS-CoV-2 promotes RIPK1 activation to facilitate viral propagation
Source: Cell Res. 2021 Oct 18;31(12):1230–43. doi: 10.1038/s41422-021-00578-7 (PMC8522117; doi:10.1038/s41422-021-00578-7)
Supplement: Supplementary file 2 — Supplementary Fig. S2 [file 41422_2021_578_MOESM2_ESM.pdf]

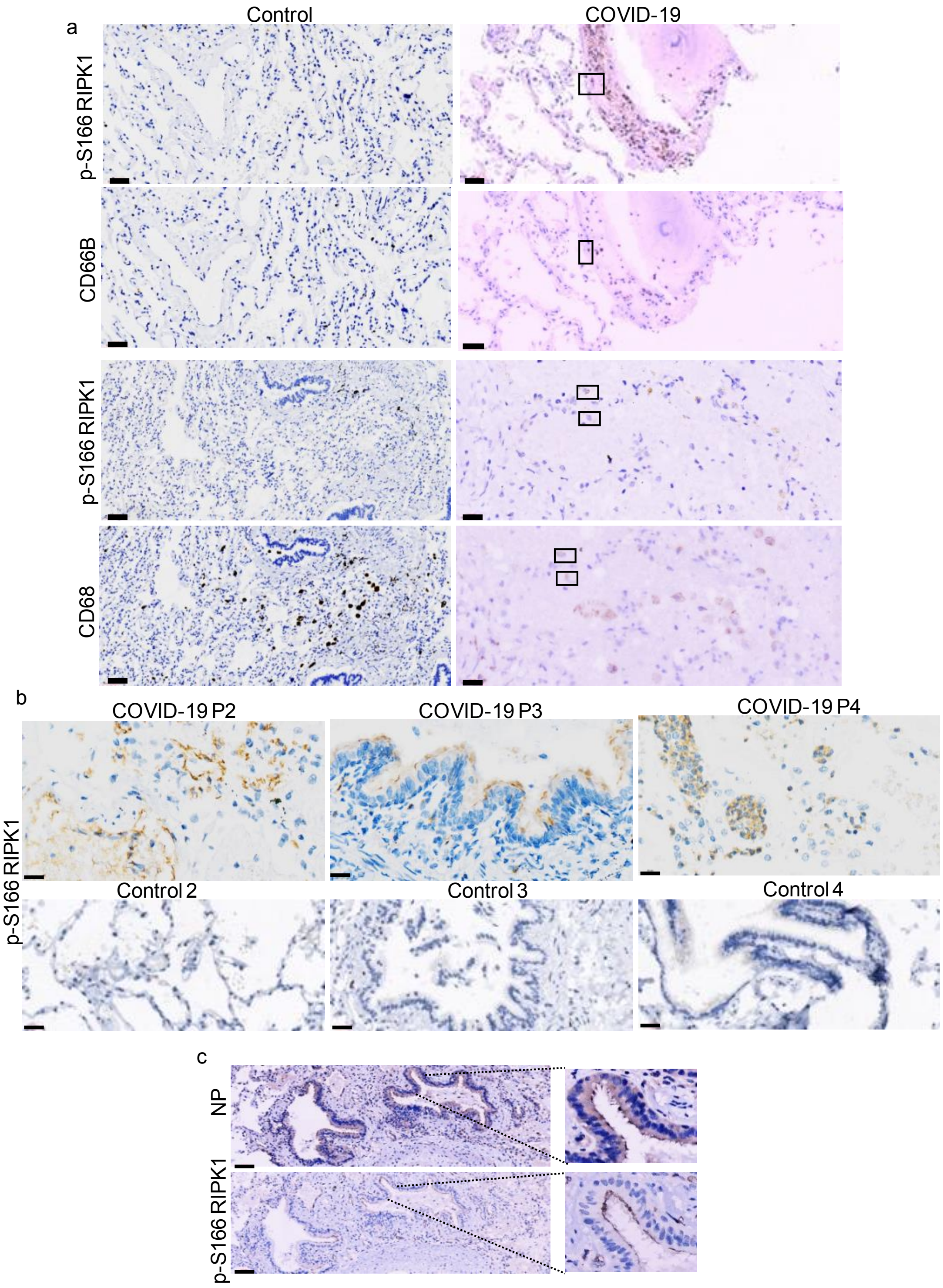

Figure S2 Activation of RIPK1 in severe COVID-19.

a. RIPK1 was activated in the neutrophils and macrophages in the lungs of severe COVID-19. The macrophage marker CD68, neutrophil marker CD66B and activated RIPK1 (p-S166 RIPK1) were

analyzed by immunohistochemistry on serial sections of severe COVID-19 and controls. Scale bars: 40 μm.

b. Immunohistochemistry of p-S166 RIPK1 in the lungs of 3 severe COVID-19 patients and 3 age-matched controls. Scale bars: 20 μm.

c. Activated RIPK1 in the SARS-CoV-2-infected ciliated bronchial epithelial cells. Immunohistochemistry staining of viral protein NP and p-S166 RIPK1 in the lungs of severe COVID-19 in adjacent consecutive serial sections. Scale bars: 20 μm. The enlarged photos show the bronchial area (right).
