# Supplementary material for: SARS-CoV-2 promotes RIPK1 activation to facilitate viral propagation
Source: Cell Res. 2021 Oct 18;31(12):1230–43. doi: 10.1038/s41422-021-00578-7 (PMC8522117; doi:10.1038/s41422-021-00578-7)
Supplement: Supplementary file 3 — Supplementary Fig. S3 [file 41422_2021_578_MOESM3_ESM.pdf]

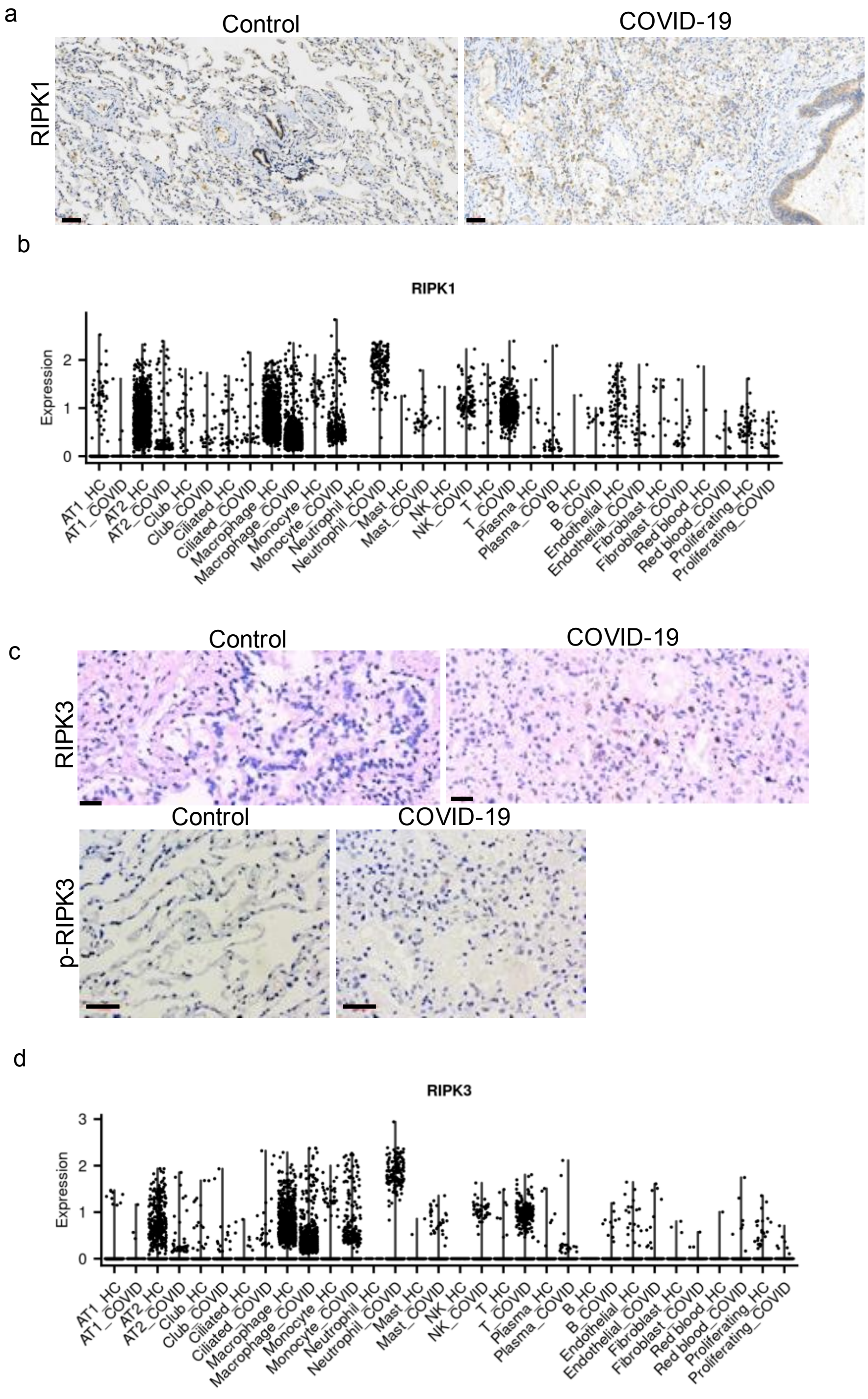

Figure S3 The expression of RIPK1 and RIPK3 in the lungs of control and COVID-19.

a-**b**. IHC (**a**) and scRNAseq analysis (**b**) of RIPK1 expression in the lungs from control and COVID-19 patients. Scale bars: 80 μm

c-**d**. Immunohistochemistry of RIPK3 (Scale bars： 20 μm) and p-RIPK3 (scale bars: 1 inch) in lung of severe COVID-19 patients and age-matched control (**c**). scRNA-seq analysis of RIPK3 expression in the lungs (**d**).
