# Supplementary material for: SARS-CoV-2 promotes RIPK1 activation to facilitate viral propagation
Source: Cell Res. 2021 Oct 18;31(12):1230–43. doi: 10.1038/s41422-021-00578-7 (PMC8522117; doi:10.1038/s41422-021-00578-7)
Supplement: Supplementary file 4 — Supplementary Fig. S4 [file 41422_2021_578_MOESM4_ESM.pdf]

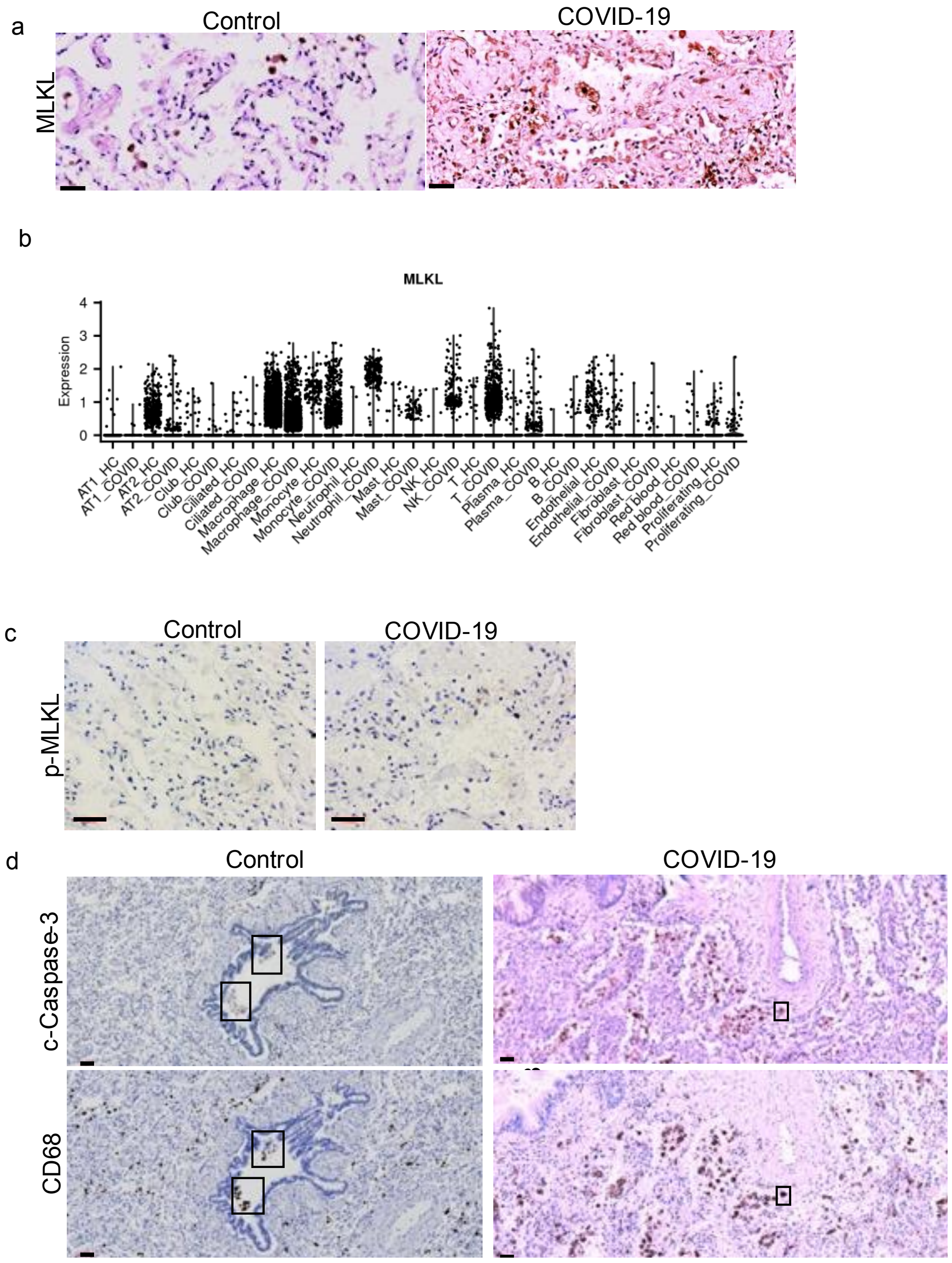

Figure S4 The expression of MLKL in the lungs of control and COVID-19

a-**b**. IHC (**a**) and scRNA-seq analysis (**b**) of MLKL expression in the lungs of control and COVID-19. Scale bars: 20 µm.

c. IHC of p-MLKL in lung of COVID-19 patient and age-matched control. Scale bars: 1 inch.

d. CD68 and c-casp3 immunohistochemical staining in the adjacent serial sections of lungs of patients and controls. Scale bars: 100 μm.
