# Supplementary material for: SARS-CoV-2 promotes RIPK1 activation to facilitate viral propagation
Source: Cell Res. 2021 Oct 18;31(12):1230–43. doi: 10.1038/s41422-021-00578-7 (PMC8522117; doi:10.1038/s41422-021-00578-7)
Supplement: Supplementary file 5 — Supplementary Fig. S5 [file 41422_2021_578_MOESM5_ESM.pdf]

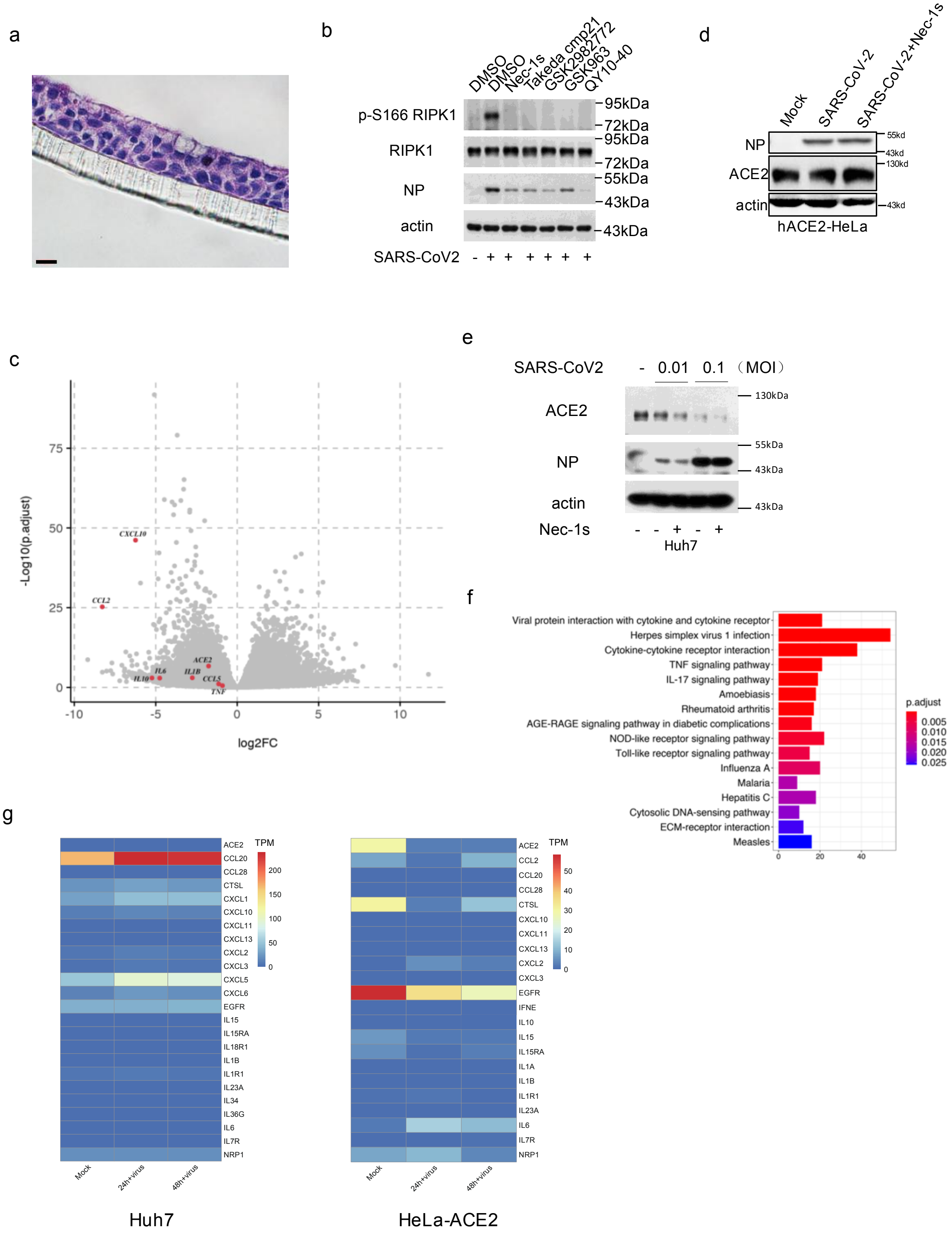

Figure S5 RIPK1 regulates the expression of ACE2 and proinflammatory cytokines in human lung organoids infected by SARS-CoV-2.

a. H&E staining of the human lung organoid to show ciliated epithelial cells in the superficial layer of the organoids. Scale bar: 100 pixels.

b. Lung organoids were first infected with SARS-CoV-2 at the MOI of 1 for 2 h, washed with PBS and replaced with fresh media with 10μM indicated RIPK1 inhibitors or vehicle and incubated for additional 48h. The cell lysates were analyzed by western blotting using indicated antibodies.

c. Volcano plot shows the differential gene expression in the human lung organoids infected by SARS-CoV-2+vehicle for 48h and SARS-CoV-2+Nec-1s for 48h. ACE2 and some of the inflammation association genes were marked.

d. HeLa-ACE2 cells were first infected with SARS-CoV-2 at the MOI of 1 for 2 h, washed with PBS and replaced with fresh media with 10μM RIPK1 inhibitors Nec-1s or vehicle and incubated for additional 48h. The cell lysates were analyzed by western blotting using indicated antibodies.

e. Huh7 cells were first infected with SARS-CoV-2 at the MOI of 0.01 or 0.1 for 2 h, washed with PBS and replaced with fresh media with 10μM RIPK1 inhibitors Nec-1s or vehicle and incubated for additional 48h. The cell lysates were analyzed by western blotting using indicated antibodies.

f. Enrichment of GO biological process (BP) terms for up-regulated genes in SARS-CoV-2-48h group compared to that of SARS-CoV-2+Nec-1s-48h group in the organoids treated as in (**b**). (adjust P value < 0.05 and absolute log2FC > 2).

g. The heatmaps show inflammation-related genes that were not induced in SARS-CoV-2 infection Huh7 cells or HeLa-ACE2 cells.
