# Supplementary material for: SARS-CoV-2 promotes RIPK1 activation to facilitate viral propagation
Source: Cell Res. 2021 Oct 18;31(12):1230–43. doi: 10.1038/s41422-021-00578-7 (PMC8522117; doi:10.1038/s41422-021-00578-7)
Supplement: Supplementary file 6 — Supplementary Fig. S6 [file 41422_2021_578_MOESM6_ESM.pdf]

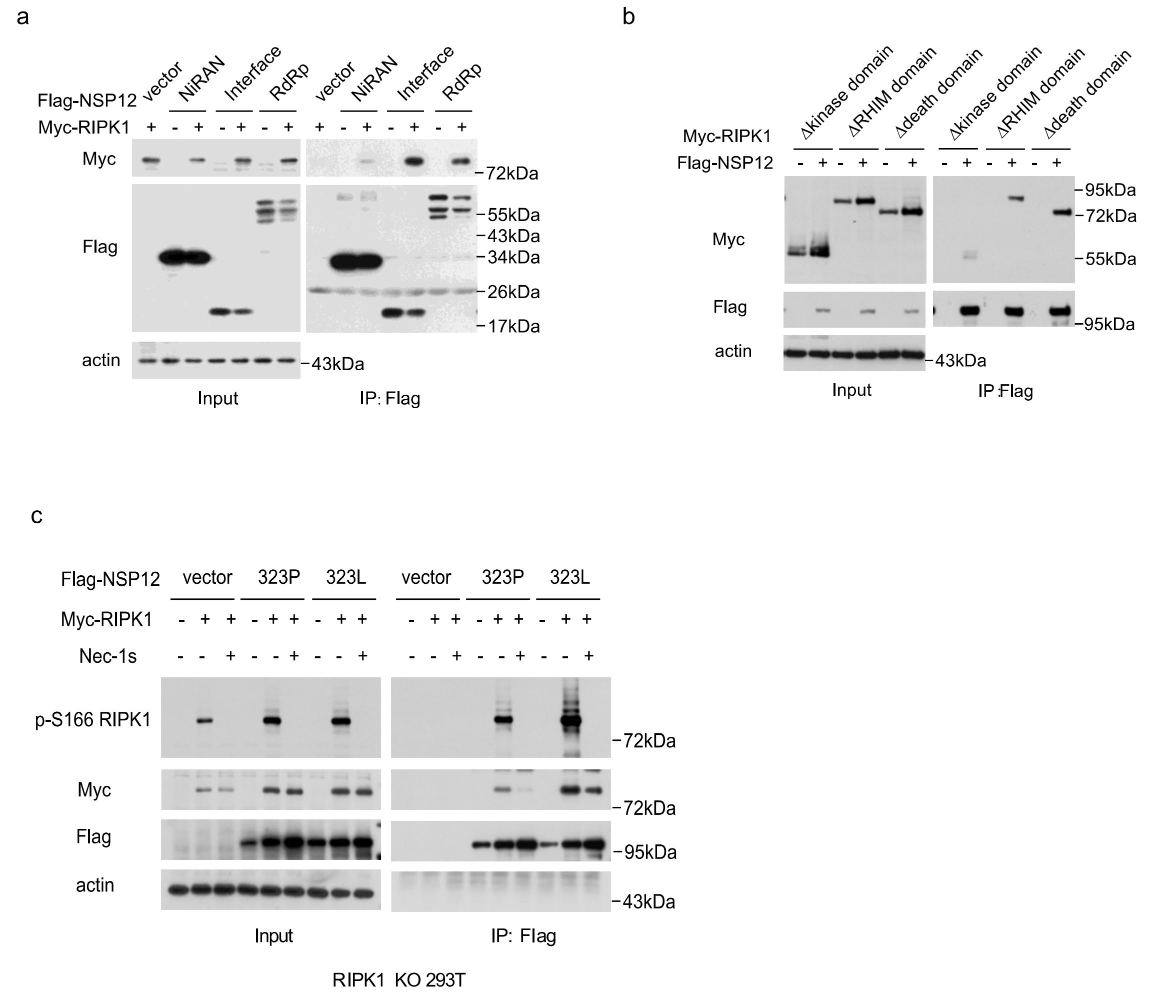

Figure S6 323L NSP12 binds RIPK1 more avidly than that of 323P NSP12.

a. RIPK1-KO 293T cells were co-transfected with expression vectors of Flag-NiRAN, Interface and RdRp domains of NSP12 and full length Myc-RIPK1as indicated and incubated for 20 h. The cells were lysed by 1% NP40 lysis buffer and immunoprecipitated with anti-Flag M2 affinity gel, and followed by analyzed by western blotting using indicated antibodies.

b. RIPK1-KO 293T cells were co-transfected with expression vectors of a full-length Flag-NSP12 and different truncations of RIPK1, including Myc-RIPK1-Δkinease domain, Myc-RIPK1-ΔRHIM domain or Myc-RIPK1-Δdeath domain as indicated and incubated for 20 h. The cell

lysates were analyzed by immunoprecipitation with anti-Flag M2 affinity gel and followed by western blotting using indicated antibodies.

c. RIPK1 KO 293T cells were co-transfected with expression vectors of Myc-RIPK1 (1μg) and vector control (1μg), Flag-323P NSP12 (1μg) or Flag-323L NSP12 (0.75μ) as indicated. Since the expression of 323L NSP12 led to higher levels of RIPK1 than that of 323P (Fig. 3e), reduced amount of 323L NSP12 expression vector was transfected to ensure the similar levels of RIPK1 and NSP12 (323L and 323P) in the cells transfected. The cells were lysed with 1% NP40 lysis buffer and the cell lysates were immunoprecipitated with anti-Flag M2 affinity gel and analyzed by western blotting using indicated antibodies.
