# Supplementary material for: SARS-CoV-2 promotes RIPK1 activation to facilitate viral propagation
Source: Cell Res. 2021 Oct 18;31(12):1230–43. doi: 10.1038/s41422-021-00578-7 (PMC8522117; doi:10.1038/s41422-021-00578-7)
Supplement: Supplementary file 7 — Supplementary Fig. S7 [file 41422_2021_578_MOESM7_ESM.pdf]

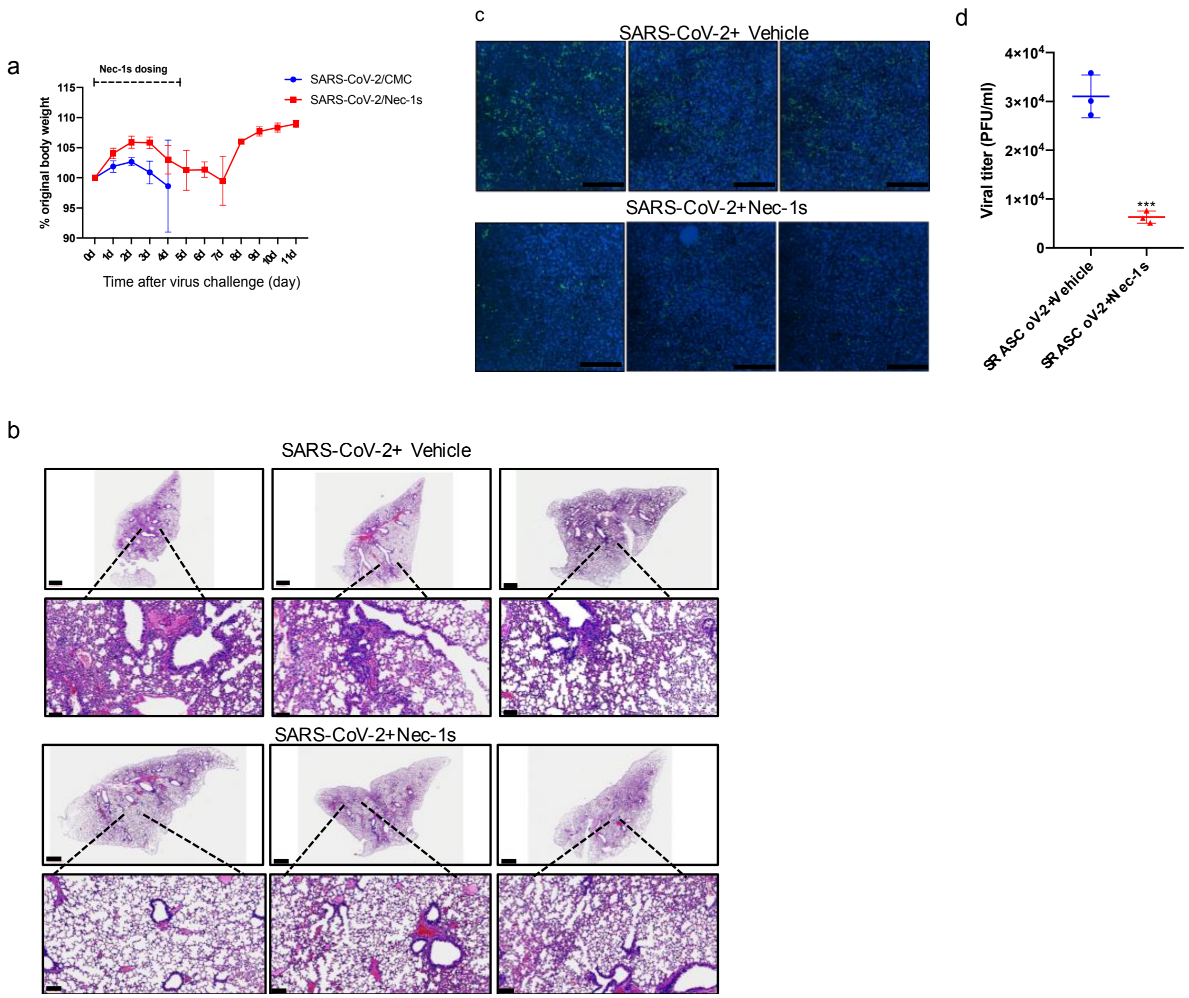

Figure S7 Nec-1s treatment can improve lung pathology and reduce lung viral load in CAG-hACE2 (AC70) transgenic mice.

a. Weight change of AC70 transgenic mice after intranasal SARS-CoV-2 infection with intragastric administration of Nec-1s or vehicle.

b. Lung pathology of the mice in the control group and Nec-1s group by H&E staining. Lungs from the two groups of mice were fixed with formalin for 48 hours and stained with hematoxylin and eosin, scale bars: 1000 um. The lower row shows the enlarged images of specified areas above. Scale bars: 100 µm. The area enlargements show obvious segmental consolidation of the lungs in the control infected AC70 transgenic mice, indicating the area with lymphocyte infiltration.

c-d. 1g lung tissue was cryogenically ground in 1ml DMEM medium. After fully grinding and centrifugation, the supernatant was collected and stored at -80℃. 4×10^4 Vero-E6 cells were seeded in 96-well plates and incubated for 12 h. Lung supernatant(50ul) was added to the cells. At 24h after the virus incubation, the SARS-CoV-2 infection was detected by immunofluorescence using COVID-19 convalescent sera (**c**). Scale bars: 1000 um. Infectious clones are automatically quantified by Cytation 5(**d**).
