# Supplementary material for: SARS-CoV-2 promotes RIPK1 activation to facilitate viral propagation
Source: Cell Res. 2021 Oct 18;31(12):1230–43. doi: 10.1038/s41422-021-00578-7 (PMC8522117; doi:10.1038/s41422-021-00578-7)
Supplement: Supplementary file 8 — Supplementary Table S1 [file 41422_2021_578_MOESM8_ESM.pdf]

Supplementary information

Table S1. Clinical data of the enrolled COVID-19 patients and healthy controls examined.

Sample number	P1	P2	P3	P4	Control 1	Control 2	Control 3	Control 4
SARS-CoV2	+	+	+	+	-	-	-	-
Severity	Severe	Severe	Severe	Severe				
Age	66	53	86	51	66	67	44	62
Gender	M	F	F	M	F	M	F	F
First symptom at onset	Fever	Fever, dyspnea	Fever, cough	Fever				
Hospitalization durations	37d	22d	5d	6d				
Other chronic basic disease	Hyperpression	NA	Atherosclerosis (IV) Heart failure, renal failure, hypertension	Hypertension				
Influenza A virus	(-)	(-)	(-)	(-)				
Influenza B virus	(-)	(-)	(-)	(-)				
Respiratory syncytial virus	(-)	(-)	(-)	(-)				
Interferon atomization	√	√	√	√				
Ribavirin	√	√	×	×				
Methylprednisolone	√	√	√	√				
CT finding	Bilaeral pneumonia	Bilaeral pneumonia	Bilaeral pneumonia	Bilaeral pneumonia	Not detected	Not detected	Not detected	Not detected
