# Supplementary material for: SARS-CoV-2 promotes RIPK1 activation to facilitate viral propagation
Source: Cell Res. 2021 Oct 18;31(12):1230–43. doi: 10.1038/s41422-021-00578-7 (PMC8522117; doi:10.1038/s41422-021-00578-7)
Supplement: Supplementary file 9 — Supplementary Table S2 [file 41422_2021_578_MOESM9_ESM.pdf]

Table S2. List of RT-qPCR **primers used in this study.**

Gene	Site	Primer
SARS-CoV2(Spike)	Foword	TGCAGGTATATGCGCTAGTTATCAG
	Reverse	CACCAAGTGACATAGTGTAGGCAAT
human ACE2	Foword	GGAGTTGTGATGGGAGTGAT
	Reverse	GATGGAGGCATAAGGATTTT
human CCL2	Foword	CAGCCAGATGCAATCAATGCC
	Reverse	TGGAATCCTGAACCCACTTCT
human CCL4	Foword	CTGTGCTGATCCCAGTGAATC
	Reverse	TCAGTTCAGTTCCAGGTCATAC
human TNF	Foword	CTCTTCTGCCTGCTGCACTTTG
	Reverse	ATGGGCTACAGGCTTGTCACTC
human IL6	Foword	CCAGCTATGAACTCCTTCTC
	Reverse	GCTTGTTCCTCACATCTCTC
human IFNB1	Foword	CTTGGATTCCTACAAAGAAGCAGC
	Reverse	TCCTCCTTCTGGAACTGCTGCA
human CXCL10	Foword	GGTGAGAAGAGATGTCTGAATCC
	Reverse	GTCCATCCTTGGAAGCACTGCA
human GAPDH	Foword	GGAGCGAGATCCCTCCAAAAT
	Reverse	GGCTGTTGTCATACTTCTCATG
human EGFR	Foword	GGCACGAGTAACAAGCTCAC
	Reverse	ATGAGGACATAACCAGCCACC
mice GAPDH	Foword	CAGGAGAGTGTTTCCTCGTCC
	Reverse	TTCCCATTCTCGGCCTTGAC
mice TNF	Foword	ACCCTCACACTCACAAACCA
	Reverse	ATAGCAAATCGGCTGACGGT
mice IL6	Foword	GCCTTCTTGGGACTGATGCT
	Reverse	TGTGACTCCAGCTTATCTCTTGG
mice CCL4	Foword	TGTGCAAACCTAACCCCGAG
	Reverse	GGGTCAGAGCCCATTGGTG
mice IFNA4	Foword	TGGCTAGGCTCTGTGCTTTC
	Reverse	CTTCAGGCAGGAAAGAGGGG
