# Supplementary material for: SARS-CoV-2 promotes RIPK1 activation to facilitate viral propagation
Source: Cell Res. 2021 Oct 18;31(12):1230–43. doi: 10.1038/s41422-021-00578-7 (PMC8522117; doi:10.1038/s41422-021-00578-7)
Supplement: Supplementary file 12 — Supplementary Video legends [file 41422_2021_578_MOESM12_ESM.pdf]

Legends of Supplementary Videos

Video S1: The video records the behavior of mice treated with CMC control after intranasal infected with SARS-CoV-2 on the third day.

Video S2: The video records the behavior of mice treated with Nec-1s after intranasal infected with SARS-CoV-2 on the third day.
